# Trends in DNA Methylation over Time Between Parous and Nulliparous Young Women

**DOI:** 10.3390/epigenomes9030024

**Published:** 2025-07-10

**Authors:** Su Chen, John W. Holloway, Wilfried Karmaus, Hongmei Zhang, S. Hasan Arshad, Susan Ewart

**Affiliations:** 1 Department of Biostatistics, College of Public Health, University of Nebraska Medical Center, Omaha, NE 68198, USA; 2 School of Human Development & Health, Faculty of Medicine, University of Southampton, Southampton SO17 1BJ, UK; j.w.holloway@soton.ac.uk; 3 NIHR Southampton Biomedical Research Centre, University Hospital Southampton NHS Foundation Trust, Southampton SO16 6YD, UK; s.h.arshad@soton.ac.uk; 4 School of Public Health, University of Memphis, Memphis, TN 38152, USA; karmaus1@memphis.edu (W.K.); hongmei.zhang@memphis.edu (H.Z.); 5 School of Clinical and Experimental Sciences, Faculty of Medicine, University of Southampton, Southampton SO17 1BJ, UK; 6 The David Hide Asthma and Allergy Research Centre, Newport PO30 5TG, Isle of Wight, UK; 7 Department of Large Animal Clinical Sciences, College of Veterinary Medicine, Michigan State University, East Lansing, MI 48824, USA; ewarts@msu.edu

**Keywords:** DNAm, CpG, gestation, pregnancy, parous, nulliparous, parturition

## Abstract

Background/Objectives: The experience of pregnancy and parturition has been associated with long-term health effects in mothers, imparting protective effects against some diseases while the risk of other diseases is increased. The mechanisms that drive these altered disease risks are unknown. This study examined DNA methylation (DNAm) changes from pre-pregnancy to several years after giving birth in parous women compared to nulliparous controls over the same time interval. Methods: Using 180 parous-associated CpGs, three analyses were carried out to test DNAm changes from pre-pregnancy at age 18 years to gestation; from gestation to post-pregnancy at age 26 years in parous women; and from 18 to 26 years in nulliparous women using linear mixed models with repeated measures. Results: The directions of DNAm changes were the same between the parous and nulliparous groups. Most CpG dinucleotides (67%, 121 of 180) had a decreasing trend while a small number (7%, 13 of 180) had an increasing trend. Of the CpGs showing increasing or decreasing DNAm, approximately half had DNAm change to a smaller extent in parous women and the other half changed more in parous women than nulliparous controls. 9% (17 of 180) changed significantly in nulliparous women only, leading to a significant difference in DNAm levels in parous women at the post-pregnancy 26 years time point. Conclusions: Pregnancy and parturition may accelerate methylation changes in some CpGs, but slow down or halt methylation changes over time in other CpGs.

## 1. Introduction

The process of bearing children has significant and lasting effects on women’s health, influencing cancer risk, metabolic health, cardiovascular health, bone health, and mental health [1,2,3,4,5,6,7]. Recent research has highlighted how pregnancy and parturition can induce lasting changes in women’s DNA methylation (DNAm) patterns [8,9]. Parous women, defined as those who have experienced at least one childbirth, exhibit distinct epigenetic profiles compared to nulliparous women (those who have not given birth) years after delivery, suggesting that reproductive history leaves a lasting molecular imprint [8,10,11]. These parous-associated methylation changes could potentially be caused by the childbearing process due to the significant hormonal, metabolic, and immunological changes during this time. For example, hormonal fluctuations during gestation, particularly in estrogen and progesterone levels, are thought to play a key role in shaping the epigenome [12,13]. The parous-associated methylation changes could also occur after delivery and be related to postpartum stress (or depression), breastfeeding, and lifestyle changes due to newborn(s) [14,15,16]. Investigating trends of methylation change from pre-pregnancy to gestation and after parturition can help better understand childbirth’s long-term effects on women’s health.

There are a small number of studies that have examined methylation around the time of pregnancy in parous women. Gruzieva et al., 2019 identified 196 CpGs that changed from before pregnancy, to during pregnancy (weeks 10 to 14 and 26 to 28 of gestation), and finally at 2 to 4 days after delivery in 21 women [17]. Lin et al., 2022 found DNAm pattern changes in 14,018 CpGs measured in each trimester of pregnancy and an average of 10 months after delivery in 10 women [9]. Fradin et al., 2023 identified 57 CpGs with methylation level changes between the first and third trimesters of pregnancy in 36 women [18]. These important studies explored the dynamics of DNAm of gestation in parous women, however, they did not include nulliparous women as controls. Without a comparison to nulliparous counterparts, it is unknown whether the methylation changes were due to factors irrelevant to pregnancy, such as a time effect. For example, 19 of the 57 CpGs identified by Fradin et al., 2023 have been associated with chronological age in a large cohort of individuals aged 14 to 94 years [18,19].

We recently identified 184 CpGs with significantly different methylation changes from age 18 (pre-pregnancy) to age 26 years (at least 6 months after delivery) between parous and nulliparous women [8]. We hypothesize that the experience of childbearing (gestation and the post-pregnancy experience) itself is the origin of these DNAm changes. In this paper, we extend our original observations by exploring the trends of methylation changes from pre-pregnancy (at 18 years) to gestation, and from gestation to post-pregnancy (at 26 years), in this subset of parous-associated CpGs. We also verified the trend (directional) changes in nulliparous women across the same ages (from 18 to 26 years) and compared our results to the published literature with the closest study designs.

## 2. Results

### 2.1. Population and Participant Characteristics

**Figure 1:**
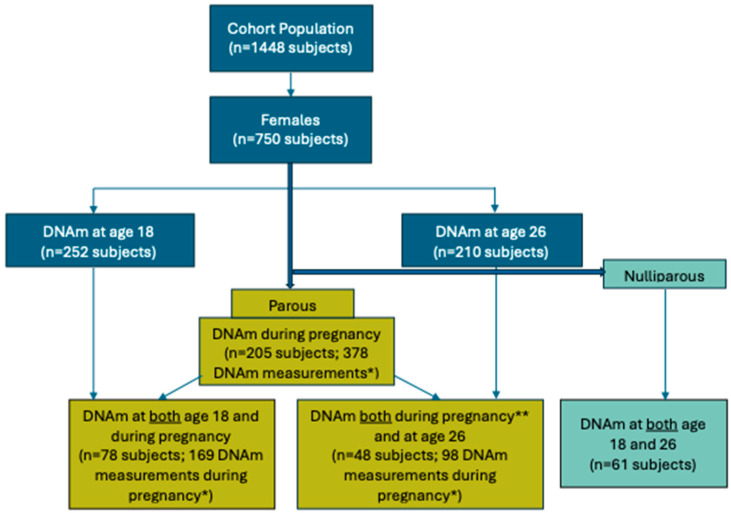
Flow diagram depicting the numbers and timings of samples collected and subsets of participants analysed in the context of the overall study design. * Some female subjects had DNAm at multiple pregnancies and up to two measurements (early and/or late gestation) of DNAm in one pregnancy (Table 2). ** DNAm data were retained only when the mother was younger than 25.5 years of age at the time of gestation.

The Isle of Wight (IOW) birth cohort contains 750 female subjects. For this study, peripheral blood samples were collected for DNAm analysis from a subset of IOW participants: 252 subjects at age 18 years (pre-pregnancy for parous women), 210 subjects at age 26 years (post-pregnancy for parous women), including both parous and nulliparous women, and 205 subjects during gestation in parous women. Figure 1 depicts how the analyzed participants were selected from the IOW study population. DNAm samples were available for 78 parous women at both age 18 and during gestation, and for 48 parous women both during gestation (when maternal age was younger than 25.5 years) and at age 26 (months to years after delivery). DNAm samples were available for 61 nulliparous women at both age 18 and 26 years. Descriptive characteristics of the IOW study population and the three analyzed samples are provided in Table 1. The characteristics, including active smoking, passive/second-hand smoking, socioeconomic status (SES), body mass index (BMI), and birth order, are covariates used in downstream analyses. Multiple DNAm measurements (378 DNAm measurements from 205 women) were collected in a subset of female subjects during gestation (Table 2).

### 2.2. Trends in DNA Methylation from Age 18 to 26 Years Between Parous and Nulliparous Women

Differential methylation changes from ages 18 to 26 years between parous women (who had not given birth before age 18 and had at least one childbirth before age 26) and nulliparous women (who had not given birth) enrolled in the IOW F1 cohort (IOW-F1) have been identified in 184 CpGs [8]. Focusing on these parous-associated CpGs, we assessed the trends of methylation changes from pre-pregnancy (age 18) to 9–39 weeks gestation, and then to post-pregnancy (age 26; 6 months to 7.5 years after delivery) in parous women and during the same time period (age 18 to 26 years) in nulliparous women in the IOW-F1 cohort. 180 out of 184 parous-associated CpGs identified in Chen et al., 2024 [8] had DNAm measurements at all three time points: pre-pregnancy, gestation, and post-pregnancy.

**Figure 2:**
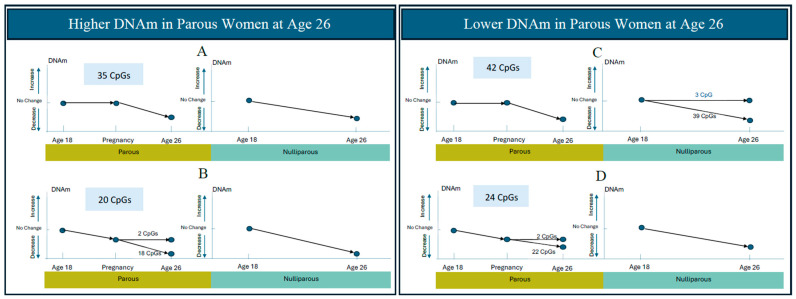
Conceptual display demonstrating decreasing directional trends and patterns in DNAm over time between parous and nulliparous subjects. CpGs in panels (**A**) (*n* = 35) and (**B**) (*n* = 20) had higher DNAm in parous women at age 26, adjusted by DNAm at age 18 years. CpGs in panels (**C**) (*n* = 42) and (**D**) (*n* = 24) had lower methylation in parous women at age 26, adjusted by DNAm at age 18 years. Note that the magnitude of DNAm change is not depicted.

Among these 180 CpGs, 121 CpGs (67%) had a trend of decreasing DNAm across the three time points (Figure 2, complete list of CpGs in Supplemental Table S1). The most common pattern was that of no change from pre-pregnancy at age 18 years to gestation and then decreased DNAm between gestation and age 26 years (Figure 2A,C). This trend was slightly more common for CpGs that had lower DNAm at age 26 in parous (vs nulliparous) women (42 CpGs, Figure 2C), as compared to those CpGs with higher DNAm in parous (vs nulliparous) women (35 CpGs, Figure 2A). Another 44 CpGs had DNAm decrease from pre-pregnancy at age 18 years to gestation (Figure 2B,D); while the level of DNAm continued to decline between gestation and age 26 years for the majority of these CpGs (18 of 20 in Figure 2B and 22 of 24 in Figure 2D), a few CpGs stabilized over this interval (2 of 20 in Figure 2B, 2 of 24 in Figure 2D). Almost the same number of CpGs in this pattern group had DNAm that was lower in parous (vs nulliparous) women at age 26 (24 CpGs, Figure 2D), as compared to those with higher DNAm in parous (vs nulliparous) women (20 CpGs, Figure 2B). Supplemental Table S1 shows the magnitude (regression coefficients in parous vs. nulliparous subjects from Chen et al., 2024 [8]) and significance (*p*-values and FDR-adjusted *p*-values) of the DNAm decreases in the 121 CpGs from Figure 2.

**Figure 3:**
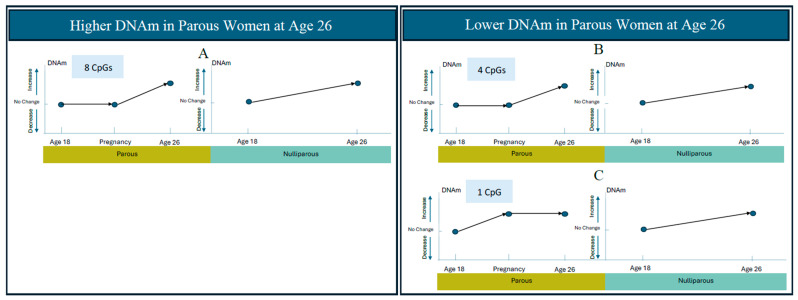
Conceptual display demonstrating increasing directional trends and patterns in DNAm over time between parous and nulliparous subjects. CpGs in panel (**A**) (*n* = 8) had higher DNAm in parous women at age 26, adjusted by DNAm at age 18 years. CpGs in panels (**B**) (*n* = 4) and (**C**) (*n* = 1) had lower methylation in parous women at age 26, adjusted by DNAm at age 18 years. Note that the magnitude of DNAm change is not depicted.

An increase in DNAm across the three time points was far less common, occurring in only 13 of 180 CpGs (7%) (Figure 3). The patterns of change included (a) increasing only during the gestation to post-pregnancy interval (12 CpG, Figure 3A,B), and (b) increasing only during the 18 years to gestation interval (1 CpG, Figure 3C). Twice as many CpGs (8 CpGs, Figure 3A) that increased only during the second interval measured (gestation to post-pregnancy) had higher DNAm in parous women (Figure 3A), as only 4 CpGs had lower DNAm in parous (Figure 3B) as compared to nulliparous women. The single CpG that increased methylation during the interval from pre-pregnancy to gestation had lower DNAm in parous as compared to nulliparous controls (Figure 3C). Table 3 shows the magnitude (regression coefficients in parous vs. nulliparous subjects from Chen et al., 2024 [8]) and significance (*p*-values and FDR-adjusted *p*-values) of the DNAm increases in the 13 CpGs from Figure 3. Negative regression coefficients between parous and nulliparous women (Supplemental Table S1 and Table 3, fifth column) indicate lower methylation in parous women (compared to nulliparous) at age 26, while positive regression coefficients (Supplemental Table S1 and Table 3, fifth column) indicate higher methylation in parous women (compared to nulliparous) at age 26.

Methylation of the remaining 46 CpGs was stable in parous women across the three time points assessed (Supplemental Figure S1, Supplemental Table S2). Most of these 46 CpGs had methylation that changed over time in nulliparous subjects, as 12 of 17 of these CpGs decreased between ages 18 to 26 years, resulting in higher DNAm in parous subjects at age 26 years (Supplemental Figure S1A), while 5 of 29 CpGs increased in nulliparous women over time, resulting in lower DNAm in parous subjects at age 26 years (Supplemental Figure S1B). Eight other CpGs changed in nulliparous subjects only, in directions not consistent with the overall DNAm trends between parous and nulliparous subjects.

Although the directions of change (increase or decrease) over time were the same for parous and nulliparous women, the magnitude of the changes differed between these groups. For example, DNAm of the 42 CpGs in Figure 2C and Supplemental Table S1 panel C did not change from age 18 to gestation, and then decreased significantly from gestation to age 26 in parous women (negative regression coefficients in the ninth column of Supplemental Table S1). The majority of these CpGs (39 of 42) also decreased significantly from age 18 to 26 years in nulliparous women (negative regression coefficients in twelfth column of Supplemental Table S1). Furthermore, when the CpGs in these two trend groups are compared to one another, they have negative regression coefficients (Supplemental Table S1, fifth column), indicating that DNAm at age 26 at these CpGs is significantly lower in parous than nulliparous women (while controlling cell-adjusted DNAm at age 18 and other confounding factors, see Chen et al., 2024 for more details [8]). Thus, we can infer that CpGs in Figure 2C and Supplemental Table S1 Panel C decrease more in parous women. By the same token, the 8 CpGs in Figure 3A and Table 3 Panel A have positive regression coefficients (Table 3, ninth, twelfth, and fifth columns), indicating that they increase to a larger magnitude in parous as compared to nulliparous women.

**Figure 4:**
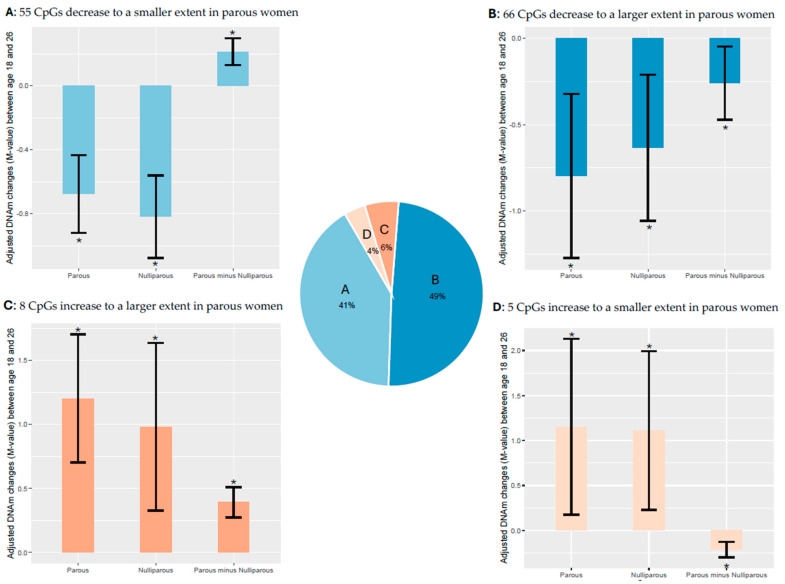
Comparison of adjusted DNAm changes from 18 to 26 years in four groups of CpGs (**A**–**D**) between parous (*n* = 28) and nulliparous (*n* = 61) subjects. Depicted are the averages (+/− one standard deviation) of the regression coefficients in the corresponding CpG groups (**A**–**D**); more specifically, the *y*-axis values for the parous and nulliparous group are DNAm changes in M-value (log2(β/(1-β)) between ages 18 and 26 adjusted by covariates specified in Section 4.5. The *y*-axis values for the “parous minus nulliparous” group are differential DNAm changes in M-value from ages 18 to 26 between parous and nulliparous subjects adjusted by covariates described in Chen et al., 2024 [8]. The regression coefficient values for the nulliparous group can be found in column 12 of Table 3 and Table S1. The regression coefficients for the parous group are calculated with the same model as the nulliparous group (See Section 4.6 for details of statistical modelings). Positive (negative) values indicate that DNAm increased (decreased) from age 18 to 26 yrs. The regression coefficients for the “parous minus nulliparous” group are obtained from Chen et al., 2024 (Table 1, column 2) [8] with positive values indicating higher DNAm in parous subjects at age 26 (DNAm at age 18 adjusted in the model). (**A**) 55 CpGs (CpGs in Figure 2A,B) decrease to a smaller extent in parous women. (**B**) 66 CpGs (CpGs in Figure 2C,D) decrease to a larger extent in parous women. (C) 8 CpGs (CpGs in Figure 3A) increase to a larger extent in parous women. (**D**) 5 CpGs (CpGs in Figure 3B,C) increase to a smaller extent in parous women. The adjusted DNAm changes between age 18 and 26 in (**D**) had large standard deviations with similar central tendency measurements between parous and nulliparous groups (*n* = 5 CpGs), thus, image (**D**) does not reflect the increase to a smaller extent in parous women. Asterisks (*) indicate significant DNAm changes with FDR-adjusted *p*-values less than 0.05.

Furthermore, among CpGs that decrease over time, roughly half of them (55 CpGs) decrease to a smaller extent in parous women (Figure 2A,B, Supplemental Tables S1A and S1B) and the other half (66 CpGs) decrease to a larger extent in parous women (Figure 2C,D, Supplemental Table S1 Panels C and D). Similarly, among CpGs that increase over time, roughly half of them (8 CpGs) increase to a larger magnitude in parous women (Figure 3A, Table 3 Panel A) and the other half (5 CpGs) increase in a smaller extent in parous women (Figure 3B,C, Table 3 Panels B and C). Thus, the different magnitudes of change between parous and nulliparous groups results in methylation differences at the endpoint of our study: DNAm at age 26, which is post-pregnancy for our parous subjects. To visualize the magnitude of changes in the four groups of CpGs that increase (or decrease) to a larger (or smaller) extent in parous women, we made boxplots of adjusted DNAm changes (M-values) from age 18 to 26 yrs in parous, nulliparous, and “parous minus nulliparous” groups (Figure 4A–D). DNAm decreased over time in the majority of CpGs (Figure 4A,B), with slightly more than half of these decreasing to a greater extent in parous women.

### 2.3. Validation and Comparison with Other Studies

**Figure 5:**
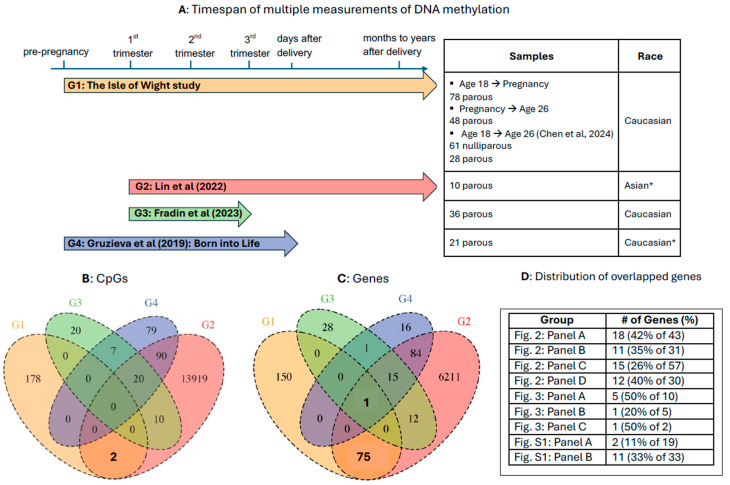
Summary of studies examining DNAm changes in parous women around the time of pregnancy. (**A**) Timespan of multiple measurements of DNAm for trajectory analyses. Our study (G1) investigated methylation changes at pre-pregnancy, during gestation, and months to years after delivery. Lin et al., 2022 [9] (G2) investigated methylation changes from the first trimester to months to years after delivery. Fradin et al., 2023 [18] (G3) assessed the methylation changes from the first to third trimesters of pregnancy. Gruzieva et al., 2019 [17] (G4) investigated methylation changes from pre-pregnancy to 2 to 4 days after delivery. * Race is inferred for the Lin and Gruziva studies as participants were enrolled at hospitals in Taiwan and Sweden, respectively. (**B**) Numbers of parous-associated CpGs that overlap across the studies. The two CpGs that overlapped between our results (G1) and Lin (G2) were cg21879513 and cg00519039. (**C**) Numbers of genes associated with the parous-associated CpGs that overlap across the studies. The one gene that overlapped across all studies was *NTRK3*. (**D**) Distribution of 76 overlapped genes among the groups of associated CpGs showing trends of increasing or decreasing DNAm across the timepoints of pre-pregnancy, gestation, and post-pregnancy (percentages of total genes in each group).

Three other independent studies have investigated the methylation trajectories during pregnancy: (i) Lin et al., 2022 [9], (ii) Fradin et al., 2023 [18], and (iii) Gruzieva et al., 2019 [17]. Figure 5A summarizes the designs of these studies in comparison to ours. Figure 5B depicts the CpGs that overlap across the four studies. Among 180 parous-associated CpGs with DNAm levels measured at pre-pregnancy, gestation, and post-pregnancy in IOW-F1, two CpGs (cg21879513 and cg00519039; Table 4) were among the 14,018 CpGs identified by Lin et al. (2022) [9]. The DNAm trend of these two CpGs was to decrease over the interval from gestation to post-pregnancy at age 26 (negative regression coefficients in Supplemental Table S1 (ninth column), panels A and D, respectively). To explore their external validation further, we analyzed the data reported in the Lin et al., 2022 [9] study by determining their mean methylation during gestation (average of methylation in beta values at 1st, 2nd, and 3rd trimesters) for cg21879513 and cg00519039 and found that the DNAm at these CpGs also decreased from gestation to their post-pregnancy sampling period, which was months to years after delivery. No CpGs overlapped with the 57 CpGs identified by Fradin et al., 2023 [18] or the 196 CpGs identified by Gruzieva et al., 2019 (Figure 5B) [17].

We also checked the numbers of genes associated with the parous-associated CpGs in each study (Figure 5C): 180 CpGs associated with 226 genes in the IOW-F1 cohort; 14,018 CpGs associated with 6398 genes in Lin et al., 2022 [9]; 57 CpGs associated with 57 genes in Fradin et al., 2023 [18]; and 196 CpGs associated with 117 genes in Gruzieva et al., 2019 [17]. There was a single gene, neurotrophic receptor tyrosine kinase 3 (*NTRK3,* associated with cg13632630), that was common to all studies. An additional 75 genes associated with the identified CpGs overlapped between our study (IOW-F1 cohort) and the Lin et al., 2022 study [9].

Among the 76 genes (associated with 67 CpGs) in common with genes associated with CpGs identified in Lin et al., 2022 [9], the majority (72%, 48 of 67) had downward trending DNAm (Table 4), while 9% (6 of 67) were upward trending (asterisks (*) mark upward trending genes in Table 3). The remaining 13 CpGs did not show a clear trend of change (asterisks (*) mark in Supplemental Table S2A,B). An additional 112 genes overlapped between Lin et al., 2022 [9] and at least one of the following studies: Gruzieva et al., 2019 [17] or Fradin et al., 2023 [18].

### 2.4. Biological Pathway Analysis 

Functional enrichment analysis of the 76 CpG-associated genes overlapping between Lin’s study and our study was carried out using the ToppFun module of ToppGene (https://toppgene.cchmc.org/, accessed on 8 June 2025) to explore KEGG pathways, diseases, and biological processes. We identified two KEGG pathways: “Aminoacyl-tRNA Biosynthesis” and “Shigella IpaB/C/D to ITGA/B-TALIN/VINCULIN signaling pathway” with FDR-adjusted *p*-value < 0.05 (Table 5). The top 10 diseases most significantly associated with these 76 genes include nervous system (including mental health) disorders and cancers (Table 6). No biological processes were identified.

We conducted the same functional enrichment analysis of the 112 genes overlapped between Lin’s and either Fradin’s or Gruzieva’s studies. One KEGG pathway, “Medicus pathogen HPV E6 to notch signaling pathway,” and 13 biological processes were identified (Supplemental Table S3). The top 10 diseases that were most significantly associated with these 112 genes were related to blood cell parameters, primarily counts of various white blood cells and platelets (Supplemental Table S4).

## 3. Discussion

Focusing on 180 parous-associated CpGs, this study evaluates the temporal changes in DNAm from pre-pregnancy (age 18), to early and/or late gestation in 78 parous women and from gestation to at least 6 months after-delivery (age 26) in 48 parous women of the IOW-F1 birth cohort in comparison to 61 nulliparous women from age 18 to 26 years in the same cohort. Of these 180 parous-associated CpGs, 46 (25.6%) were characterized by methylation that remained stable across the three time points of before-, during-, and after-pregnancy in parous women, while 121 (90% of the remaining 134) decreased across the same time intervals in parous women as well as from 18 to 26 years in nulliparous women. This agrees with existing studies reporting a tendency for overall DNAm to decrease over time [20]. A much smaller number of CpGs (13, 10% of 134) had methylation increase from age 18 to gestation and post-pregnancy (age 26) in parous women and also increased from age 18 to 26 years in nulliparous controls.

Of the CpGs showing a trend change (either increasing or decreasing DNAm), approximately half had DNAm change to a smaller extent in parous women (Figure 4A,D). Furthermore, 9% (17 of 180) changed significantly in nulliparous women only (Supplemental Figure S1; 12 CpGs in panel A and 5 CpGs in panel B), leading to a significant difference in DNAm levels as compared to parous women at the 26 year time point. These findings offer an additional explanation that, rather than changes being induced by pregnancy, aspects of parturition and the post-pregnancy process may slow down or halt the methylation changes (either increases or decreases) that otherwise occur over time in some CpGs.

Next, CpGs with different trends in DNAm changes may be driven by different factors. This is supported by our results in which some CpGs (Figure 2B,D and Figure 3C; Supplemental Table S1 Panels B and D, Table 3 Panel C) change significantly from pre-pregnancy to gestation, with most going on to further change significantly from gestation to post-pregnancy (only 5 of 45 CpGs remain unchanged in this second time interval), which may be due to the rise of pregnancy-related hormones such as estrogen and progesterone. Furthermore, methylation changes from gestation to post-pregnancy may reflect the different drivers of the methylation changes. For example, the CpGs that remain unchanged from pre-pregnancy (age 18) to gestation, and then change significantly from gestation to post-pregnancy (age 26) (Figure 2A,C and Figure 3A,B) may be related to factors associated with parturition, e.g., rises in 17β-estradiol, oxytocin, and prostaglandins along with withdrawal of progesterone, and/or the postpartum period, e.g., postnatal stress, sleep disruption, and nursing.

These findings have been further validated by comparing our study to other studies of methylation changes in parous women by Lin et al., 2022 [9], Fradin et al., 2023 [18], and Gruzieva et al., 2019 [17] (Figure 5). All of the four studies (including ours) focused on uncomplicated pregnancies and blood methylation changes. The participants in Fradin et al., 2023 [18], Gruzieva et al., 2019 [17] and our study were mostly Caucasians and in Lin et al., 2022 [9] were Asians (Taiwanese). Our study and Gruzieva et al., 2019 [17] collected DNAm data at a pre-pregnancy time point, while the others did not. Our study and Lin et al., 2022 [9] collected DNAm data months to years after childbirth; in contrast, DNAm was available for only a few days after delivery in the Gruzieva et al., 2019 [17] studies. Fradin et al., 2023 [18] only collected DNAm data during pregnancy. Despite these study design and methodological differences, our study had two CpGs (cg00519039 and cg21879513) and 76 genes that overlapped with the results reported by Lin et al., 2022 [9] (Figure 5B,C). One of these genes (*NTRK3*) was reported in all four studies (Figure 5C).

The Rho GTPase activating protein 19 gene (*ARHGAP19*) is associated with cg00519039, one of two CpGs that overlapped between our results and the Lin study. This gene encodes regulators of Rho GTPases, which are involved in cell migration, proliferation, differentiation, and G1 cell cycle progression [21]. This CpG is also associated with the FRAT regulator of WNT signaling pathway 1 gene (*FRAT1*). *FRAT1* (frequently rearranged in advanced T-cell lymphomas) positively regulates the WNT signaling pathway and may function in tumor progression and lymphomagenesis [22]. The other CpG identified by both studies, cg21879513, is associated with the *COL20A1* gene encoding the alpha-1 chain of type XX collagen, which appears in cartilage. The *COL20A1* gene has been associated with a variety of conditions, including pulmonary fibrosis [23], palmoplantar keratoderma [24], diabetic kidney disease [25], and several cancers [26]. Interestingly, DNAm of *COL20A1* was associated with a microbiota taxon that influences gut metabolism in adults during behavioral weight loss treatment [27] and also with polycyclic aromatic hydrocarbons (PAHs) exposure among non-smokers [28].

Neurotrophic receptor tyrosine kinase 3 (NTRK3) was the one gene that overlapped with all studies (Figure 4C), based on its associated CpG (cg13632630, Supplemental Table S1 panel B). NTRK3 has been found to be frequently involved in gene fusion events underlying soft tissue neoplasms [29,30,31], in which these gene fusions alter kinase activity. More specifically, ETV6-NTRK3 fusions are present in various hematopoietic and epithelial neoplasms [32,33,34], including secretory breast carcinoma [35].

The top diseases associated with the 76 genes we identified to overlap between Lin et al., 2022 [9] and our data were related to mental health (e.g., schizophrenia, ADHD), brain neoplasia (e.g., glioblastoma) and other neoplasias (e.g., fibrosarcoma, colorectal carcinoma) (Table 6). These 76 overlapped genes were also associated with two KEGG biological pathways: (1) Aminoacyl-tRNA Biosynthesis and (2) Shigella IpaB/C/D to ITGA/B-TALIN/VINCULIN signaling pathway (Table 5). The biosynthesis of aminoacyl-tRNA synthases may be related to the altered disease risk seen in parous women later in life stemming from the biosynthesis and regulatory roles of these enzymes. Aminoacyl-tRNA synthases play a key step in ensuring the proper amino acid sequence during translation of RNA into protein. In addition, they have recently been shown to be involved in immune cell development and signaling, and thus are increasingly recognized as having a role in infectious disease, autoimmune disease, tumor immunity, and neurological disease [36,37,38,39]. The connection between later life disease risk modifications among parous women may seem less obviously related to Shigella signaling, however, in a recent systematic review of 62 studies on gut microbiota composition in autoimmune neurologic diseases, Deng et al. found that an increase in Escherichia-Shigella was found in patients with autoimmune encephalitis, neuromyelitis optica spectrum disorders, myasthenia gravis, and multiple sclerosis [40].

The overlapped CpGs and genes between Lin et al., 2022 [9] and our study may capture post-pregnancy changes that are due to reasons like postnatal stress and sleep disruption. This finding was supported by our further investigation of the two overlapped CpGs, as well as the biological pathways, biological processes, and top diseases associated with 76 overlapped genes. It should be noted that the sample size of Lin et al., 2022 [9] was too small (*n* = 10) to withstand multiple testing adjustments and thus relatively large numbers of CpGs with raw *p*-values < 0.001 were reported. This may have contributed to the higher level of overlapping results with ours and the other two comparison studies. We also observed overlapping results among the three comparison studies (and not shared by ours) as Lin et al., 2022 [9] had 120 overlapped CpGs and 112 overlapped genes (some CpGs had no genes reported in the manifest file) with either Fradin et al., 2023 or Gruzieva et al., 2019 [17] (Figure 5C). These 112 overlapping genes were associated with the KEGG pathway “Medicus pathogen HPV E6 to notch signaling pathway” and 13 biological processes mainly in cell differentiation and cell adhesion (Supplemental Table S3). The top diseases associated with the 112 genes were related to the immune system such as leukocyte and platelet counts (Supplemental Table S4).

Two critical design elements shared by the three comparison studies that differed from ours were: (1) they investigated methylation changes in parous women only, while our work has a nulliparous group for comparison; and (2) they did not extend as far into the pre-pregnancy (months to years prior) and/or post-pregnancy (months to years after) periods as ours did. Thus, the DNAm changes of the overlapped CpGs among the three studies may be temporary due to reasons like hormone changes, returning to pre-pregnancy values soon after delivery. Our study was intentionally designed to ignore temporarily changing CpGs, and focus instead on the CpGs that change from the pre-pregnancy (months to years prior) and/or post-pregnancy (months to years after) periods in parous women compared to nulliparous women in the same time interval.

While our study is the largest in the current literature, it is still relatively small in terms of the number of subjects (28 parous and 61 nulliparous women with DNAm at both ages 18 and 26 years). Our sample size allowed us to identify 180 parous-associated CpGs [8], however, we may have missed some CpGs with smaller effect sizes, or we could have identified false positives. To assess the risk for this, we conducted a power analysis, which indicated we have 80% power to detect a medium effect size 0.5 with 27 subjects in each group and a significance level of 1.5 $\times \;10^{-7}$ (after Bonferroni correction) using linear mixed modeling with repeated measures. While these 180 CpGs may not reflect those with the smallest effect size, our focus on parous-associated CpGs is a strength because we avoid random, temporal changes that are not related or are only temporarily related to gestation. Next, our statistical analyses were carried out in two steps (changes from pre-pregnancy to gestation, and then changes from gestation to post-pregnancy) in parous women with different sets of subjects (only 9 subjects had DNAm at all three time points) to maximize the sample size at each analysis. We included all 78 women with DNAm both at age 18 and during gestation, including mothers older than 25.5 years when pregnant (52 mothers were age 18–25.4 years and 44 mothers were age 25.5–42 years at pregnancy; some mothers had pregnancies both before and after 25.5 years). To additionally test the validity of this approach, we performed two sensitivity analyses: (1) DNAm changes from pre-pregnancy to gestation, and gestation to post-pregnancy with 9 subjects that had DNAm at all three time points; and (2) DNAm changes from pre-pregnancy to gestation with all mothers younger than 25.5 years when pregnant. Similar coefficient directions (positive or negative) were observed in significant CpGs in both analyses. Furthermore, the second sensitivity analysis indicated that the trend changes may be similar between mothers with pregnancy at different ages, say 24 vs. 29 years. However, women in our cohort study are likely too young to formally investigate whether the mother’s age at pregnancy contributes to changing methylation trends. Third, while we identified similar decreasing and increasing DNAm trends in parous and nulliparous women on the same CpGs, we did not collect DNAm data between ages 18 and 26 years in nulliparous women and thus did not have a time point comparable to when parous women were pregnant. Thus, we had to assume a linear trend in our modeling. Finally, there are currently no other independent cohort studies with DNAm at all three of our time points (before, during, and months to years after pregnancy) and in nulliparous controls for a formal validation analysis. Thus, the three studies we used for validation each had some, but not all, of these elements in their study design and that may be why our findings were only partially validated.

## 4. Materials and Methods

### 4.1. Study Population

The IOW birth cohort is a longitudinal, population-based study established in the United Kingdom to investigate the developmental origins and natural progression of asthma, allergic diseases, and related chronic conditions [41]. The study initially enrolled expectant parents (first generation, IOW-F0) between 1989 and 1990 while they were expecting the second generation (IOW-F1). As the IOW-F1 participants reached adulthood, female members were further monitored during their pregnancies (2011–2015), giving rise to the third generation (IOW-F2). The IOW-F1 cohort has been prospectively followed up for 26 years, in which a subset of parous female participants have been followed up months to years after delivery at age 26. The cohort is predominantly white, with 98% of the F1 generation identifying as such.

### 4.2. Parous Status

Participants’ parous or nulliparous status up to age 26 years was assessed by their participation (or not) in the third generation IOW study as well as from the medical records. Female participants were classified as nulliparous up to age 26 years if they met the following criteria: (1) no DNAm data collected during pregnancy, (2) no child enrolled in the IOW-F2 cohort, and (3) no documented history of pregnancy, childbirth, early pregnancy termination, infertility treatment, or late miscarriage based on IOW study records or linked medical data.

### 4.3. DNA Methylation Measurements, Processing, and Quality Control

Peripheral whole blood samples were collected at three time points: age 18 years, during 9–39 weeks gestation for parous women, and age 26 years. DNA extraction was performed using either a standard salting procedure [42] or commercial kits (Qiagen, Germantown, MD, USA) with DNA concentration determined by fluorometry (Qubit, Invitrogen™, Thermo Fisher Scientific, Waltham, MA, USA). To assess epigenome-wide methylation, age 18 and gestation samples were assayed using either Illumina Infinium HumanMethylation450 arrays or Infinium MethylationEPIC BeadChips (Illumina, Inc., San Diego, CA, USA), while all the age 26 year samples were processed exclusively on MethylationEPIC BeadChips. Multiple identical control samples were assigned to each bisulfite conversion batch to assess assay variability. DNAm data preprocessing was conducted using the CPACOR pipeline [43], including (1) background correction and quantile normalization using the minfi R package (v1.36.0) [44]; (2) calculation of beta (β) values using quantile-normalized intensities from (1), defined as proportions of intensity of methylated (M) over the sum of methylated and unmethylated (U) sites/probes (β = M/[c + M + U], where c is a constant to prevent zero in the denominator if M + U is too small); (3) conversion to M-values (logit-transformed β-values) for downstream statistical analysis [45]; and (4) quality control to exclude (i) probes with detection *p*-value > 10^−16^ in at least 95% samples, (ii) samples with poor detection (*p*-value > 10^−16^) in >95% of CpGs [43], and (iii) sex chromosome CpGs. The use of both HumanMethylation450 (containing > 450 K CpGs) and MethylationEPIC (containing > 850 K CpGs) platforms is a consequence of technology evolution as HumanMethylation450 arrays are no longer available. To account for potential technical variation, DNAm data also underwent batch correction using the ComBat algorithm in R, which adjusts for platform differences and processing batches while preserving biological variability [46].

### 4.4. Cell Estimation

It has been shown that DNAm varies substantially between cell types [47,48]. Prior studies suggested accounting for the cell-type composition of samples when analyzing DNAm data [49,50,51]. Our DNAm was analyzed in whole blood and thus we adjusted for cell composition of whole blood while assessing DNAm changes. Proportions of the cell types CD4 + T-cells, CD8 + T-cells, natural killer cells, B-cells, neutrophils, monocytes, and eosinophils were estimated through the estimateCellCounts function from the R package minfi and were then included in the analyses as confounders [44].

### 4.5. Confounding Variables

For the DNAm changes from age 18 years (reference group) to during gestation, the following confounding variables were adjusted in the model: age, gestational weeks when DNAm were collected during pregnancy (set zero for age 18 years), active and passive smoking, socio-economic status (SES), birth order (set zero for age 18 years), and cell composition. Body mass index (BMI) during pregnancy did not reflect a woman’s realistic BMI and thus was not included in the analyses involving DNAm during pregnancy. For the DNAm changes from gestation (reference group) to age 26, similar confounding variables including age, gestational weeks when DNAm were collected during pregnancy (set zero for age 26 data), active and passive smoking, SES, and cell composition. The number of children mothers had by age 26 (i.e., their birth order) was not collected by the study and thus not included in the model. For the DNAm changes from age 18 (reference group) to age 26 in nulliparous women, we adjusted for age, active and passive smoking, SES, BMI, and cell composition as confounding variables.

Active smoking was recorded as “yes” for a current smoker. Second-hand smoking was recorded as “yes” if anyone in the household smokes inside the home. SES categories at each time point were generated separately by K-mean clustering using education, housing information, and income, which were extracted from the corresponding questionnaires. Education was recorded as “School,” “6th Form College,” “Further Education,” and “Other.” Housing information at age 18 and 26 years was recorded in ordinal data as “Rented Council/Housing Assoc.,” “Rented Private,” “Lives with Parents,” “Owned Private,” and “Other.” Housing information during pregnancy was recorded as “number of rooms in the house.” Income was also recorded in the ordinal groups as “Less than £12,000,” “£12,000 to £17,999,” “£18,000 to £29,999,” “£30,000 to £41,999,” “Greater than £42,000,” and “Prefer not to say.” In the cluster analysis, education and housing were recoded as ordinal integers from the worst to the best. “Other” in education and housing information, and “Prefer not to say” in income were treated as missing values. Clusters were chosen based on R2, Pseudo-F, and CCC statistics. Four SES clusters (1, worst; 4, best) were identified for ages 18 and 26 and five clusters (1, worst; 5, best) for during pregnancy. See Table 1 for detailed interpretation of the clusters.

### 4.6. Statistical Analysis

Focusing on the 184 CpGs that were differentially methylated between parous and nulliparous women at age 26 years [8], we examined the trends of methylation changes before (age 18 years), during gestation, and post-pregnancy (age 26 years) in parous women. The upward/downward trends were also validated in nulliparous women during the same period (at ages 18 and 26 years). Among the 184 CpGs, 180 CpGs have DNAm data at all three timepoints and thus were included in our analyses. To identify CpG sites that were significantly differentially methylated, three linear mixed models with repeated measures were implemented, each adjusting for confounding variables as described in Section 4.5. The models compared DNA methylation levels between the following time points: (1) age 18 years (reference group) and gestation, (2) gestation (reference group) and age 26 years, and (3) age 18 years (reference group) and age 26 years in nulliparous women only. Repeated measures per subject were utilized to assess random subject effects reflected by random intercepts in the linear mixed models. Addressing random subject effects helps improve statistical power. Only overlapping individuals in both time points were used in the corresponding analysis. Multiple testing was adjusted by controlling the false discovery rate (FDR) at 0.05 for each set of analyses [52]. We then grouped the 180 CpGs according to their changing trends in parous and nulliparous women.

Next, we compared the findings with three other independent studies that investigated the methylation trajectories during pregnancy: Lin et al., 2022 [9], Fradin et al., 2023 [18], and Gruzieva et al., 2019 [17], focusing on overlapping CpGs and genes. We checked the consistency of trend changes for overlapped CpGs and performed gene enrichment analysis in ToppFun (https://toppgene.cchmc.org/enrichment.jsp, accessed on 8 June 2025) to identify significant biological processes, KEGG biological pathways, and diseases associated with the overlapped genes.

## 5. Conclusions

In conclusion, our results identified trends in DNAm changes associated with childbearing status in women. Furthermore, pregnancy may not only accelerate methylation changes in CpGs, but also slow down or halt methylation changes. Some CpGs that change significantly during pregnancy may be temporary, while other CpGs that remain unchanged or change in a smaller magnitude during pregnancy (compared to nulliparous women) may be affected by pregnancy. Diseases associated with the CpGs and genes we identified involved cancers, the nervous system, and substance uses. In particular, the methylation on CpG sites on the *ARHGAP19* and *NTRK3* genes, identified in all four studies, promise potential explanation of disease risk variation in nulliparous and parous women. Additional studies are needed in cohorts with follow up years to decades after childbirth to investigate whether and how these parous-associated DNAm changes are related to health outcomes later in life. We also need to explore whether one’s age at pregnancy affects the trend changes from pre-pregnancy to gestation and post-pregnancy in the long run. Finally, our results suggest that there is a need to include nulliparous women as a reference while investigating the pattern of DNAm changes due to pregnancy and childbirth.

## Figures and Tables

**Table 1:** Descriptive characteristics of the cohort population and subsets of analyzed samples.

Characteristic	Measurement Time	Status	Cohort Population (%) *	Analyzed Parous Samples: Age 18→ Gestation (%) **	Analyzed Parous Samples: Gestation → Age 26 (%) ***	Analyzed Nulliparous Samples: Age 18 → Age 26 (%) ****
Active Smoking	Age 18	Y (%)	159 (29%)	25 (39%)	-	10 (19%)
		N (%)	388 (71%)	39 (61%)	-	44 (81%)
	Pregnancy	Y (%)	178 (32%)	54 (32%)	40 (41%)	-
		N (%)	385 (68%)	115 (68%)	58 (59%)	-
	Age 26	Y (%)	176 (31%)	-	20 (42%)	20 (33%)
		N (%)	385 (69%)	-	28 (58%)	41 (67%)
Passive Smoking	Age 18	Y (%)	438 (67%)	67 (86%)	-	43 (70%)
		N (%)	215 (33%)	11 (14%)	-	18 (30%)
	Pregnancy	Y (%)	171 (34%)	40 (24%)	38 (39%)	-
		N (%)	330 (66%)	125 (76%)	60 (61%)	-
	Age 26	Y (%)	148 (26%)	-	15 (31%)	18 (30%)
		N (%)	412 (74%)	-	33 (69%)	43 (70%)
Socioeconomic Status (SES)	Age 18	1: low education, low housing, low income (%)	130 (20%)	16 (21%)	-	13 (21%)
		2: low education, low housing, high income (%)	269 (42%)	23 (29%)	-	29 (48%)
		3: high education, low housing, medium income (%)	122 (19%)	20 (26%)	-	14 (23%)
		4: high education, high housing, high income (%)	124 (19%)	19 (24%)	-	5 (8%)
	Pregnancy	1: low education, low # rooms, low income (%)	77 (19%)	23 (19%)	20 (21%)	-
		2: low to medium education, low # rooms, low income (%)	97 (24%)	36 (29%)	29 (30%)	-
		3: high education, low # room, low to medium income (%)	114 (29%)	41 (33%)	25 (26%)	-
		4: low to medium education, high # rooms, medium income (%)	61 (15%)	16 (13%)	15 (16%)	-
		5: medium education, high # rooms, high income (%)	51 (13%)	8 (6%)	7 (7%)	-
	Age 26	1: low education, low housing, low income (%)	85 (30%)	-	21 (44%)	9 (15%)
		2: low education, low housing, high income (%)	34 (12%)	-	7 (15%)	6 (10%)
		3: medium education, high housing, low income (%)	63 (22%)	-	6 (13%)	16 (26%)
		4: high education, low housing, medium income (%)	65 (23%)	-	13 (27%)	18 (30%)
		5: medium education, high housing, high income (%)	38 (13%)	-	1 (2%)	12 (20%)
Body Mass Index (BMI)	Age 18	Mean (SD)	24 (11.6)	-	-	23 (4.0)
	Age 26	Mean (SD)	27(6.8)	-	-	26 (5.5)
Birth Order	Pregnancy	1 (%)	233 (57%)	61 (52%)	56 (58%)	-
		2 (%)	126 (31%)	41 (35%)	28 (29%)	-
		3 (%)	38 (9%)	10 (8%)	9 (9%)	-
		4+ (%)	12 (3%)	6 (5%)	3 (3%)	-

* The cohort population contained 750 female subjects; percentages were calculated based on non-missing observations for each characteristic. ** 78 subjects with samples at both age 18 years and during gestation, with up to 169 data points per characteristic due to multiple measurements from one pregnancy and/or multiple pregnancies; percentages calculated based on non-missing observations for each characteristic. *** 48 subjects with samples at both during gestation (when the mother was younger than 25.5 years of age at the time of gestation) and age 26 years, with up to 98 data points per characteristic due to multiple measurements from one pregnancy and/or multiple pregnancies; percentages were calculated based on non-missing observations for each characteristic. **** 61 nulliparous subjects; percentages were calculated based on non-missing observations for each characteristic. # indicates “number of”.

**Table 2:** Descriptive characteristics of the DNA methylation measurements during gestation in the cohort population and subsets of analyzed samples.

		Study Population (%) *	Analyzed Samples (Age 18→ Gestation) (%) **	Analyzed Samples (Gestation → Age 26) (%) ***
Number of pregnancies with DNAm per mother	1	143 (70%)	42 (54%)	34 (70%)
	2	53 (26%)	30 (38%)	13 (27%)
	3	8 (4%)	5 (6%)	1 (2%)
	4	1 (1%)	1 (1%)	0 (0%)
Number of DNAm measurements per pregnancy	1	176 (64%)	73 (60%)	28 (44%)
	2	101 (36%)	48 (40%)	35 (56%)
Gestational age (weeks)	Mean (SD)	21.5 (8.3)	21.3 (8.3)	21.4 (8.5)
	Range	8~40	9~39	9~39
Mother’s age at pregnancy (years)	Mean (SD)	24.4 (3.4)	25 (3.6)	22.8 (1.6)
	Range	18~40	17~38	18~25

* Of the 750 females in the cohort population, 205 subjects had 378 gestational DNAm measurements; percentages were calculated based on non-missing observations (out of the 205 female subjects) for each characteristic. ** 78 subjects with samples at both age 18 years and during gestation, with up to 169 data points per characteristic due to multiple measurements from one pregnancy and/or multiple pregnancies; percentages were calculated based on non-missing observations for each characteristic. *** 48 subjects with samples at both during gestation (when the mother was younger than 25.5 years of age at the time of gestation) and age 26 years, with up to 98 data points per characteristic due to multiple measurements from one pregnancy and/or multiple pregnancies; percentages were calculated based on non-missing observations for each characteristic.

**Table 3:** Magnitude and significance indicators of CpGs with DNA methylation that increase across the timepoints of pre-pregnancy (age 18), gestation (for parous subjects), and post-pregnancy (age 26) between parous and nulliparous subjects.

Panel	CpG	Chr	Gene Name	Chen et al., 2024 [8] (Parous vs. Nulliparous)	Parous (Pre-Pregnancy to Gestation)			Parous (Gestation to Post- Pregnancy)			**Nulliparous** **(Age 18 to Age 26)**		
				Coef (FDRp) **	Coef	*p*-Value	FDR-p	Coef	*p*-Value	**FDR-p**	**Coef**	***p*-Value**	**FDR-p**
A	cg13600489	14	*NKX2-1*	0.51 (5 $\times \;10^{-4}$)	−0.025	0.882	0.912	0.423	2.2 $\times \;10^{-2}$	3.1 $\times \;10^{-2}$	0.307	1.8 $\times \;10^{-2}$	2.1 $\times \;10^{-2}$
	cg24693287	1	*SERINC2*	0.50 (6 $\times \;10^{-4}$)	0.435	0.097	0.206	0.799	7.2 $\times \;10^{-6}$	1.9 $\times \;10^{-5}$	0.741	1.4 $\times \;10^{-6}$	2.1 $\times \;10^{-6}$
	cg27549834	3	MYRIP *	0.28 (7 $\times \;10^{-4}$)	0.236	0.336	0.495	0.716	1.3 $\times \;10^{-5}$	3.1 $\times \;10^{-5}$	0.808	2.3 $\times \;10^{-9}$	4.5 $\times \;10^{-9}$
	cg20824761	15	PAQR5 *	0.53 (9 $\times \;10^{-4}$)	0.431	0.569	0.692	1.746	4.4 $\times \;10^{-4}$	8.7 $\times \;10^{-4}$	1.865	1.2 $\times \;10^{-8}$	2.1 $\times \;10^{-8}$
	cg24737639	12	NUP37 *; C12orf48 *	0.37 (1 $\times \;10^{-3}$)	0.187	0.691	0.776	1.206	7.3 $\times \;10^{-4}$	1.4 $\times \;10^{-3}$	1.417	5.5 $\times \;10^{-8}$	9.3 $\times \;10^{-8}$
	cg25074185	11	PHOX2A *	0.45 (1 $\times \;10^{-3}$)	0.895	0.086	0.194	0.843	1.5 $\times \;10^{-2}$	2.3 $\times \;10^{-2}$	1.886	2.4 $\times \;10^{-9}$	4.6 $\times \;10^{-9}$
	cg09754845	7	*MICALL2;* *UNCX*	0.29 (2 $\times \;10^{-3}$)	0.123	0.453	0.586	0.309	2.9 $\times \;10^{-2}$	4.1 $\times \;10^{-2}$	0.316	3.1 $\times \;10^{-3}$	3.8 $\times \;10^{-3}$
	cg27395066	17	*ACBD4*	0.22 (4 $\times \;10^{-3}$)	0.254	0.186	0.309	0.685	2.6 $\times \;10^{-4}$	5.3 $\times \;10^{-4}$	0.508	5.6 $\times \;10^{-5}$	7.3 $\times \;10^{-5}$
B	cg11236850	4	ACOX3 *	−0.19 (9 $\times \;10^{-4})$	0.362	0.101	0.208	0.846	4.2 $\times \;10^{-6}$	1.2 $\times \;10^{-5}$	1.135	1.5 $\times \;10^{-17}$	5.9 $\times \;10^{-17}$
	cg25418406	17	*RANGRF;* *SLC25A35*	−0.30 (1 $\times \;10^{-3}$)	0.787	0.103	0.208	0.712	1.2 $\times \;10^{-2}$	1.9 $\times \;10^{-2}$	2.480	2.6 $\times \;10^{-15}$	7.4 $\times \;10^{-15}$
	cg25734490	12	*ASCL1*	−0.28 (3 $\times \;10^{-3})$	0.456	0.063	0.157	0.376	2.8 $\times \;10^{-2}$	3.9 $\times \;10^{-2}$	1.221	3.0 $\times \;10^{-13}$	7.5 $\times \;10^{-13}$
	cg26316702	2	*TEKT4*	−0.09 (6 $\times \;10^{-3}$)	0.102	0.039	0.128	0.140	4.0 $\times \;10^{-3}$	6.3 $\times \;10^{-3}$	0.138	1.1 $\times \;10^{-4}$	1.4 $\times \;10^{-4}$
C	cg10773016	4	*BLOC1S4;* KIAA0232 *	−0.21 (3 $\times \;10^{-3}$)	0.561	9.0$\times \;10^{-4}$	6.8$\times \;10^{-3}$	0.149	0.223	0.257	0.580	2.5 $\times \;10^{-7}$	4.0 $\times \;10^{-7}$

Coef, regression coefficient; FDR-p, false discovery rate adjusted *p*-value. * Overlapped genes between our study and Lin et al., 2022 [9]. ** Regression coefficients of DNAm at age 26 on parous status (yes/no) and cell-adjusted DNAm at age 18 and other covariates (see Chen et al., 2024 for more details [8]). Note: The sum of regression coefficients in columns 6 and 9 is not comparable to coefficients in column 12 to quantify differential methylation changes between parous and nulliparous women; column 5 reports the comparison of differential methylation changes between parous and nulliparous in the same model while adjusting for appropriate covariates.

**Table 4:** Magnitude and significance indicators of 47 CpGs with DNAm that decrease across the timepoints of pre-pregnancy (age 18), during gestation (for parous subjects), and post-pregnancy (age 26) between parous and nulliparous subjects.

Panel	CpG	Chr	Gene Name	Chen et al., 2024 [8] (Parous vs. Nulliparous)	Parous (Age 18 to Gestation)			Parous (Gestation to Age 26)			**Nulliparous** **(Age 18 to Age 26)**		
				Coef (FDRp) *	Coef	*p*-Value	FDR-p	Coef	*p*-Value	**FDR-p**	**Coef**	***p*-Value**	**FDR-p**
A	cg04413148	16	*CTRL*	0.16 (5 $\times \;10^{-4}$)	−0.186	0.145	0.266	−0.683	4.4 $\times \;10^{-10}$	3.7$\times \;10^{-9}$	−0.900	1.4$\times \;10^{-33}$	3.5$\times \;10^{-32}$
	cg25364469	3	*ZBTB20*	0.18 (9 $\times \;10^{-4}$)	−0.159	0.095	0.205	−0.310	9.5 $\times \;10^{-5}$	2.0$\times \;10^{-4}$	−0.788	2.5$\times \;10^{-26}$	3.0$\times \;10^{-25}$
	cg17675386	10	*RGS10*	0.17 (9 $\times \;10^{-4}$)	0.022	0.820	0.869	−0.544	9.9 $\times \;10^{-10}$	6.8$\times \;10^{-9}$	−0.685	1.7$\times \;10^{-17}$	6.7$\times \;10^{-17}$
	cg14312661	11	*CARS*	0.14 (9 $\times \;10^{-4}$)	−0.139	0.039	0.128	−0.707	1.2 $\times \;10^{-19}$	6.9$\times \;10^{-18}$	−0.951	3.4$\times \;10^{-39}$	1.3$\times \;10^{-37}$
	cg00050271	16	*CMIP*	0.29 (9 $\times \;10^{-4}$)	−0.207	0.084	0.192	−0.515	1.5 $\times \;10^{-5}$	3.6$\times \;10^{-5}$	−0.944	1.0$\times \;10^{-22}$	8.8$\times \;10^{-22}$
	cg20009923	12	*ATP2B1*	0.29 (1 $\times \;10^{-3}$)	−0.042	0.792	0.844	−0.535	3.3 $\times \;10^{-5}$	7.4$\times \;10^{-5}$	−0.768	4.9$\times \;10^{-11}$	1.0$\times \;10^{-10}$
	cg20368567	17	*NF1; EVI2A*	0.30 (1 $\times \;10^{-3}$)	−0.079	0.613	0.720	−0.617	5.2 $\times \;10^{-7}$	1.9$\times \;10^{-6}$	−0.867	9.8$\times \;10^{-16}$	3.1$\times \;10^{-15}$
	cg00243040	7	*SND1;* *MIR129-1;* *LEP*	0.19 (1 $\times \;10^{-3}$)	−0.066	0.425	0.571	−0.312	5.8 $\times \;10^{-5}$	1.3$\times \;10^{-4}$	−0.500	8.0$\times \;10^{-14}$	2.1$\times \;10^{-13}$
	cg10705060	3	*BFSP2*	0.33 (1 $\times \;10^{-3}$)	−0.158	0.374	0.521	−0.755	1.1 $\times \;10^{-6}$	3.6$\times \;10^{-6}$	−1.273	4.0$\times \;10^{-16}$	1.3$\times \;10^{-15}$
	cg08557624	6	*FARS2*	0.28 (2 $\times \;10^{-3}$)	−0.172	0.257	0.406	−0.298	3.5 $\times \;10^{-2}$	4.9$\times \;10^{-2}$	−0.679	2.5$\times \;10^{-8}$	4.3$\times \;10^{-8}$
	cg03972656	18	*SETBP1*	0.22 (2 $\times \;10^{-3}$)	−0.227	0.034	0.120	−0.700	1.4 $\times \;10^{-10}$	1.3$\times \;10^{-9}$	−0.886	1.5$\times \;10^{-17}$	5.9$\times \;10^{-17}$
	cg03626857	19	*ZNF227*	0.24 (2 $\times \;10^{-3}$)	−0.069	0.655	0.751	−0.525	3.8 $\times \;10^{-5}$	8.4$\times \;10^{-5}$	−0.553	7.4$\times \;10^{-9}$	1.4$\times \;10^{-8}$
	cg08285768	15	*AKAP13*	0.27 (2 $\times \;10^{-3}$)	−0.413	0.062	0.157	−0.829	3.8 $\times \;10^{-6}$	1.1$\times \;10^{-5}$	−0.941	2.4$\times \;10^{-11}$	5.3$\times \;10^{-11}$
	cg06944982	8	*PTK2*	0.33 (2 $\times \;10^{-3}$)	−0.103	0.616	0.720	−0.761	4.1 $\times \;10^{-6}$	1.2$\times \;10^{-5}$	−0.899	2.2$\times \;10^{-10}$	4.5$\times \;10^{-10}$
	cg23033749	7	*ST7*	0.10 (2 $\times \;10^{-3}$)	−0.160	0.045	0.136	−0.159	1.1 $\times \;10^{-2}$	1.6$\times \;10^{-2}$	−0.205	3.9$\times \;10^{-6}$	5.7$\times \;10^{-6}$
	cg21879513	20	*COL20A1*	0.15 (2 $\times \;10^{-2}$)	0.081	0.567	0.692	−0.313	2.0 $\times \;10^{-2}$	2.9$\times \;10^{-2}$	−0.396	2.3$\times \;10^{-4}$	2.9$\times \;10^{-4}$
	cg26436731	1	*SPMIP3;* *ZBTB18*	0.10 (2 $\times \;10^{-2}$)	−0.174	0.038	0.128	−0.606	7.0 $\times \;10^{-12}$	7.9$\times \;10^{-11}$	−0.722	7.3$\times \;10^{-20}$	3.8$\times \;10^{-19}$
B	cg02133624	3	*DLG1;* *DLG1-AS1*	0.16 (1 $\times \;10^{-4}$)	−0.310	1.3 $\times \;10^{-6}$	4.0$\times \;10^{-5}$	−0.294	1.3 $\times \;10^{-6}$	4.1$\times \;10^{-6}$	−0.705	5.9$\times \;10^{-23}$	5.3$\times \;10^{-22}$
	cg19035181	20	*NINL; NANP;* *GINS1*	0.14 (6 $\times \;10^{-4}$)	−0.307	1.4 $\times \;10^{-4}$	1.8$\times \;10^{-3}$	−0.496	5.5 $\times \;10^{-10}$	4.0$\times \;10^{-9}$	−0.885	2.1$\times \;10^{-30}$	3.7$\times \;10^{-29}$
	cg11003536	11	*PRDM10;* *LINC00167*	0.18 (9 $\times \;10^{-4}$)	−0.341	2.0 $\times \;10^{-4}$	2.3$\times \;10^{-3}$	−0.750	1.0 $\times \;10^{-15}$	2.3$\times \;10^{-14}$	−0.983	1.7$\times \;10^{-29}$	2.5$\times \;10^{-28}$
	cg13632630	15	*LINC00052; NTRK3*	0.16 (9 $\times \;10^{-4}$)	−0.174	4.2 $\times \;10^{-3}$	2.32$\times \;10^{-2}$	−0.251	1.1 $\times \;10^{-5}$	2.7$\times \;10^{-5}$	−0.497	5.5$\times \;10^{-19}$	2.8$\times \;10^{-18}$
	cg18777774	17	*ABR; BHLHA9*	0.14 (9 $\times \;10^{-4}$)	−0.524	3.7 $\times \;10^{-8}$	1.7$\times \;10^{-6}$	−0.783	4.1$\times \;10^{-17}$	1.1$\times \;10^{-15}$	−1.209	3.9$\times \;10^{-40}$	2.3$\times \;10^{-38}$
	cg08166720	17	*ZZEF1*	0.25 (2 $\times \;10^{-3}$)	−0.410	1.1 $\times \;10^{-2}$	4.62$\times \;10^{-2}$	−0.788	1.5 $\times \;10^{-9}$	9.9$\times \;10^{-9}$	−1.192	1.1$\times \;10^{-18}$	5.2$\times \;10^{-18}$
	cg18909525	9	*ASB6*	0.30 (2 $\times \;10^{-3}$)	−0.412	3.3 $\times \;10^{-5}$	5.3$\times \;10^{-4}$	−0.330	2.5 $\times \;10^{-3}$	4.1$\times \;10^{-3}$	−0.883	2.4$\times \;10^{-15}$	7.1$\times \;10^{-15}$
	cg00697880	3	*OSBPL10*	0.19 (2 $\times \;10^{-3}$)	−0.366	1.0 $\times \;10^{-3}$	7.2$\times \;10^{-3}$	−0.735	4.5 $\times \;10^{-13}$	6.7$\times \;10^{-12}$	−1.033	5.4$\times \;10^{-20}$	3.0$\times \;10^{-19}$
	cg00335252	2	*RBMS1*	0.17 (3 $\times \;10^{-3}$)	−0.422	2.6 $\times \;10^{-9}$	1.5$\times \;10^{-7}$	−0.433	5.9 $\times \;10^{-9}$	3.4$\times \;10^{-8}$	−0.891	7.0$\times \;10^{-27}$	9.0$\times \;10^{-26}$
C	cg08653258	3	*BHLHE40;* *ARL8B*	−0.19 (9 $\times \;10^{-4}$)	−0.253	0.013	0.051	−0.402	2.9 $\times \;10^{-8}$	1.5$\times \;10^{-7}$	−0.737	3.1$\times \;10^{-20}$	1.9$\times \;10^{-19}$
	cg17672798	10	*ADARB2*	−0.17 (9 $\times \;10^{-4}$)	−0.178	0.148	0.266	−0.485	5.8 $\times \;10^{-8}$	2.6$\times \;10^{-7}$	−0.745	1.3$\times \;10^{-21}$	8.4$\times \;10^{-21}$
	cg21533331	19	*AC002116.7; THAP8; WDR62*	−0.19 (9 $\times \;10^{-4}$)	−0.066	0.588	0.708	−0.220	3.2 $\times \;10^{-2}$	4.5$\times \;10^{-2}$	−0.208	1.6$\times \;10^{-3}$	1.9$\times \;10^{-3}$
	cg00647046	10	*INPP5A;* *CFAP46*	−0.34 (1 $\times \;10^{-3}$)	0.055	0.772	0.829	−0.531	1.5 $\times \;10^{-3}$	2.6$\times \;10^{-3}$	−0.661	1.4$\times \;10^{-7}$	2.4$\times \;10^{-7}$
	cg26328510	10	*CUGBP2*	−0.56 (1 $\times \;10^{-3}$)	−0.061	0.715	0.794	−1.054	3.4 $\times \;10^{-6}$	1.0$\times \;10^{-5}$	−0.857	3.9$\times \;10^{-6}$	5.7$\times \;10^{-6}$
	cg14575222	9	*NAIF1;* *SLC25A25*	−0.18 (2 $\times \;10^{-3}$ )	−0.144	0.184	0.309	−4.79	3.3 $\times \;10^{-7}$	1.3$\times \;10^{-6}$	−0.770	1.7$\times \;10^{-18}$	7.8$\times \;10^{-18}$
	cg22279507	2	*FARSB*	−0.30 (2 $\times \;10^{-3}$)	−0.246	0.058	0.156	−0.739	5.2 $\times \;10^{-10}$	4.0$\times \;10^{-9}$	−0.747	6.9$\times \;10^{-11}$	1.5$\times \;10^{-10}$
	cg14594063	10	*ADAM12*	−0.27 (2 $\times \;10^{-3}$)	−0.297	0.063	0.157	−0.412	1.9 $\times \;10^{-3}$	3.3$\times \;10^{-3}$	−0.880	6.8$\times \;10^{-14}$	1.8$\times \;10^{-13}$
	cg03964554	17	*RAD51C;* *PPM1E*	−0.17 (3 $\times \;10^{-3}$)	0.085	0.395	0.535	−0.532	3.3 $\times \;10^{-9}$	2.0$\times \;10^{-8}$	−0.281	5.8$\times \;10^{-5}$	7.4$\times \;10^{-5}$
	cg26319015	7	*ACTB; FSCN1*	−0.28 (3 $\times \;10^{-3}$)	−0.181	0.385	0.526	−0.788	3.0 $\times \;10^{-5}$	6.9$\times \;10^{-5}$	−0.588	6.3$\times \;10^{-6}$	9.1$\times \;10^{-6}$
	cg01832012	7	*TPK1*	−0.14 (2 $\times \;10^{-2}$)	−0.153	0.083	0.192	−0.348	7.9 $\times \;10^{-6}$	2.0$\times \;10^{-5}$	−0.354	5.9$\times \;10^{-8}$	1.0$\times \;10^{-7}$
	cg03029734	6	*GRIK2*	−0.12 (5 $\times \;10^{-2}$)	0.008	0.928	0.938	−0.282	7.7$\times \;10^{-4}$	1.4$\times \;10^{-3}$	−0.252	1.4$\times \;10^{-5}$	2.0$\times \;10^{-5}$
D	cg08870757	17	*ALOX12*	−0.320 (4 $\times \;10^{-4}$)	−0.451	2.5 $\times \;10^{-5}$	4.5 $\times \;10^{-4}$	−0.845	3.3 $\times \;10^{-15}$	6.0$\times \;10^{-14}$	−0.820	1.7$\times \;10^{-15}$	5.2$\times \;10^{-15}$
	cg22789605	12	*SLC11A2*	−0.207 (6 $\times \;10^{-4}$)	−0.433	3.4 $\times \;10^{-6}$	8.7 $\times \;10^{-5}$	−0.294	1.1 $\times \;10^{-3}$	2.0$\times \;10^{-3}$	−0.573	9.3$\times \;10^{-15}$	2.6$\times \;10^{-14}$
	cg15210276	19	*HAPLN4*	−0.183 (7 $\times \;10^{-4}$)	−0.306	4.3 $\times \;10^{-4}$	3.4 $\times \;10^{-3}$	−0.470	4.9 $\times \;10^{-8}$	2.2$\times \;10^{-7}$	−0.715	1.3$\times \;10^{-18}$	5.9$\times \;10^{-18}$
	cg19681610	1	*NOS1AP*	−0.155 (9 $\times \;10^{-4}$)	−0.290	1.3 $\times \;10^{-3}$	8.6 $\times \;10^{-3}$	−0.568	1.7 $\times \;10^{-12}$	2.2$\times \;10^{-11}$	−0.768	3.4$\times \;10^{-25}$	3.4$\times \;10^{-24}$
	cg08288130	8	*DOK2*	−0.255 (9 $\times \;10^{-4}$)	−0.402	5.4 $\times \;10^{-5}$	8.1 $\times \;10^{-4}$	−0.506	5.5 $\times \;10^{-8}$	2.5$\times \;10^{-7}$	−0.710	2.0$\times \;10^{-15}$	6.2$\times \;10^{-15}$
	cg01788221	16	*ANKRD11*	−0.106 (9 $\times \;10^{-4}$)	−0.392	1.3 $\times \;10^{-6}$	4.0 $\times \;10^{-5}$	−0.830	6.1 $\times \;10^{-28}$	1.1$\times \;10^{-25}$	−1.039	4.1$\times \;10^{-45}$	3.7$\times \;10^{-43}$
	cg09043104	8	*LINC00536;* *EIF3H*	−0.118 (1 $\times \;10^{-3}$)	−0.338	8.1 $\times \;10^{-6}$	1.8 $\times \;10^{-4}$	−0.372	9.6 $\times \;10^{-7}$	3.2$\times \;10^{-6}$	−0.650	1.9$\times \;10^{-22}$	1.6$\times \;10^{-21}$
	cg00519039	10	*ARHGAP19;* *FRAT1*	−0.201 (2 $\times \;10^{-3}$)	−0.293	7.0 $\times \;10^{-3}$	3.1 $\times \;10^{-2}$	−0.476	5.2 $\times \;10^{-7}$	1.9$\times \;10^{-6}$	−0.619	5.5$\times \;10^{-13}$	1.3$\times \;10^{-12}$
	cg13676583	5	*DDX41; DOK3*	−0.131 (3 $\times \;10^{-3}$)	−0.200	1.3 $\times \;10^{-3}$	8.6 $\times \;10^{-3}$	−0.368	1.1 $\times \;10^{-9}$	7.3$\times \;10^{-9}$	−0.476	9.0$\times \;10^{-16}$	2.9$\times \;10^{-15}$
	cg16419756	5	*SLC12A8*	−0.094 (1 $\times \;10^{-2}$)	−0.224	2.6 $\times \;10^{-4}$	2.4 $\times \;10^{-3}$	−0.537	1.3$\times \;10^{-15}$	2.6$\times \;10^{-14}$	−0.736	1.1$\times \;10^{-30}$	2.2$\times \;10^{-29}$

Coef, regression coefficient; FDR-p, false discovery rate adjusted *p*-value. * Regression coefficients of DNAm at age 26 on parous status (yes/no) and cell-adjusted DNAm at age 18 and other covariates (see Chen et al., 2024 for more details [8]). Note: The sum of regression coefficients in columns 6 and 9 is not comparable to coefficients in column 12 to quantify differential methylation changes between parous and nulliparous women; column 5 reports the comparison of differential methylation changes between parous and nulliparous in the same model while adjusting for appropriate covariates.

**Table 5:** KEGG biological pathway associated with 76 overlapped genes between our study and Lin et al., 2022 [9].

KEGG Biological Pathway	Source	*p*-Value	*q*-Value FDR B&H	Hit Count in Query (Hit Count in Genome)	Hits in the Query List
Shigella IpaB/C/D to ITGA/B-TALIN/VINCULIN signaling pathway	KEGG Medicus Pathways	6.29 $\times \;10^{-4}$	0.03	2 (9)	*ACTB*, *PTK2*
Aminoacyl-tRNA Biosynthesis	KEGG Legacy Pathways	6.97 $\times \;10^{-4}$	0.03	3 (41)	*CARS1*, *FARSB*, *FARS2*

**Table 6:** Top 10 diseases associated with 76 overlapped genes between our study and Lin et al., 2022 [9].

Disease Name	*p*-Value	*q*-Value FDR B&H	Hit Count in Query (Hit Count in Genome)	Hits in the Query List
Schizophrenia	3.23 $\times \;10^{-7}$	0.001	13 (883)	*CELF2*, *INPP5A*, *NTRK3*, *ACTB*, *NFASC*, *DLG1*, *GRIK2*, *CTRL*, *ADAM12*, *BHLHE40*, *ALOX12*, *LEP*, *NOS1AP*
Attention deficit hyperactivity disorder, substance abuse, antisocial behaviour measurement	5.55 $\times \;10^{-6}$	0.004	11 (801)	*PHOX2A*, *ZBTB20*, *CELF2*, *SND1*, *UNC5B*, *NTRK3*, *SETBP1*, *NINL*, *NFASC*, *PARPBP*, *PTK2*
Fibrosarcoma	1.91 $\times \;10^{-5}$	0.010	2 (3)	*NF1*, *NTRK3*
Glioblastoma	5.01 $\times \;10^{-5}$	0.016	4 (79)	*NF1*, *GRIK2*, *BHLHE40*, *PTK2*
Susceptibility to shingles measurement	5.52 $\times \;10^{-5}$	0.016	4 (81)	*TPK1*, *ZKSCAN3*, *MYRIP*, *NFASC*
Giant Cell Glioblastoma	6.37 $\times \;10^{-5}$	0.016	4 (84)	*NF1*, *GRIK2*, *BHLHE40*, *PTK2*
Colorectal Carcinoma	7.19 $\times \;10^{-5}$	0.016	9 (702)	*ACAP1*, *TCF3*, *SLC11A2*, *NF1*, *SETBP1*, *PPM1E*, *ZKSCAN3*, *EIF3H*, *ADARB2*
Risk-taking Behaviour	1.36 $\times \;10^{-4}$	0.020	9 (764)	*ZBTB20*, *NF1*, *SND1*, *NUP37*, *NTRK3*, *ZKSCAN3*, *NFASC*, *PARPBP*, *ARHGAP19*
APOE carrier status, cerebral amyloid angiopathy	1.69 $\times \;10^{-4}$	0.020	3 (42)	*FZR1*, *SETBP1*, *GRIK2*
Glioblastoma Multiforme	1.88 $\times \;10^{-4}$	0.020	4 (111)	*NF1*, *GRIK2*, *BHLHE40*, *PTK2*

## Data Availability

Information on data access is available from this link: https://allergyresearch.org.uk/studies/birth-cohort/#cohort-data-use.

## Supplementary Materials

The following supporting information can be downloaded at: https://www.mdpi.com/article/10.3390/epigenomes9030024/s1.

## Abbreviations

The following abbreviations are used in this manuscript:

BMI	Body mass index
CpG	Cytosine-phosphate-Guanine dinucleotide
DNAm	DNA methylation
FDR	False discovery rate
IOW	Isle of Wight
SES	Socioeconomic status
