# Supplementary material for: Trends in DNA Methylation over Time Between Parous and Nulliparous Young Women
Source: Epigenomes. 2025 Jul 10;9(3):24. doi: 10.3390/epigenomes9030024 (PMC12286171; doi:10.3390/epigenomes9030024)
Supplement: Supplementary file 1 [file epigenomes-09-00024-s001.zip › epigenomes-3664546-supplementary.pdf]

Table S1: Magnitude and significance indicators of all CpGs with DNA methylation that decrease across the timepoints of pre-pregnancy (age 18), gestation (for parous subjects), and post-pregnancy (age 26) between parous and nulliparous subjects.

Panel	CpG	Chr	Gene Name	Chen et al., 2024 (Parous vs Nulliparous)	Parous (Age 18 to Pregnancy)			Parous (Pregnancy to Age 26)			Nulliparous (Age 18 to Age 26)		
				Coef(FDRp)**	Coef	p-value	FDR-p	Coef	p-value	FDR-p	Coef	p-value	FDR-p
A	cg08934660	3	XIRP1	0.22(4E-04)	-0.123	0.118	0.230	-0.353	4.8E-07	1.8E-06	-0.648	4.1E-20	2.4E-19
	cg04413148	16	CTRL*	0.16(5E-04)	-0.186	0.145	0.266	-0.683	4.4E-10	3.7E-09	-0.900	1.4E-33	3.5E-32
	cg22029297	1	LIN28	0.21(7E-04)	-0.066	0.350	0.508	-0.447	1.6E-08	8.7E-08	-0.741	1.0E-21	7.0E-21
	cg25667998	19	ZSCAN5B	0.15(7E-04)	-0.200	0.021	0.077	-0.253	7.4E-04	1.4E-03	-0.669	3.9E-18	1.7E-17
	cg25364469	3	ZBTB20*	0.18(9E-04)	-0.159	0.095	0.205	-0.310	9.5E-05	2.0E-04	-0.788	2.5E-26	3.0E-25
	cg17675386	10	RGS10*	0.17(9E-04)	0.022	0.820	0.869	-0.544	9.9E-10	6.8E-09	-0.685	1.7E-17	6.7E-17
	cg14312661	11	CARS*	0.14(9E-04)	-0.139	0.039	0.128	-0.707	1.2E-19	6.9E-18	-0.951	3.4E-39	1.3E-37
	cg00050271	16	CMIP*	0.29(9E-04)	-0.207	0.084	0.192	-0.515	1.5E-05	3.6E-05	-0.944	1.0E-22	8.8E-22
	cg25138283	3	GPR149; MME	0.24(1E-03)	-0.296	0.055	0.151	-0.534	3.8E-06	1.1E-05	-0.829	1.6E-18	7.5E-18
	cg20009923	12	ATP2B1*	0.29(1E-03)	-0.042	0.792	0.844	-0.535	3.3E-05	7.4E-05	-0.768	4.9E-11	1.0E-10
	cg20368567	17	NF1*;EVI2A	0.30(1E-03)	-0.079	0.613	0.720	-0.617	5.2E-07	1.9E-06	-0.867	9.8E-16	3.1E-15
	cg20616821	1	MAGI3	0.19(1E-03)	-0.087	0.364	0.516	-0.528	4.8E-08	2.2E-07	-0.862	3.3E-22	2.6E-21
	cg00243040	7	SND1*; MIR129-1; LEP*	0.19(1E-03)	-0.066	0.425	0.571	-0.312	5.8E-05	1.3E-04	-0.500	8.0E-14	2.1E-13
	cg24481506	10	LOC619207	0.33(1E-03)	-0.254	0.064	0.157	-0.536	2.5E-04	5.1E-04	-0.938	4.6E-22	3.3E-21
	cg03502215	3	LOC440945	0.28(1E-03)	-0.250	0.141	0.264	-0.703	7.4E-06	1.9E-05	-1.022	2.2E-16	7.8E-16
	cg10705060	3	BFSP2*	0.33(1E-03)	-0.158	0.374	0.521	-0.755	1.1E-06	3.6E-06	-1.273	4.0E-16	1.3E-15
	cg16750953	5	TERT	0.27(1E-03)	-0.047	0.774	0.829	-0.492	1.2E-04	2.5E-04	-0.777	2.4E-10	4.8E-10
	cg11813009	15	CTDSPL2	0.26(1E-03)	0.151	0.496	0.628	-0.779	1.9E-05	4.5E-05	-0.839	3.1E-08	5.4E-08
	cg22535729	8	RP1	0.25(1E-03)	-0.169	0.300	0.454	-0.581	7.0E-04	1.3E-03	-0.996	3.4E-11	7.4E-11
	cg08557624	6	FARS2*	0.28(2E-03)	-0.172	0.257	0.406	-0.298	3.5E-02	4.9E-02	-0.679	2.5E-08	4.3E-08
	cg14683065	10	LRRC27	0.54(2E-03)	-0.241	0.104	0.208	-1.320	5.5E-10	4.0E-09	-1.903	1.4E-25	1.5E-24
	cg17807001	17	C1QTNF1	0.15(2E-03)	-0.137	0.169	0.295	-0.708	2.3E-14	3.7E-13	-0.986	1.8E-31	3.9E-30
	cg03972656	18	SETBP1*	0.22(2E-03)	-0.227	0.034	0.120	-0.700	1.4E-10	1.3E-09	-0.886	1.5E-17	5.9E-17
	cg03626857	19	ZNF227*	0.24(2E-03)	-0.069	0.655	0.751	-0.525	3.8E-05	8.4E-05	-0.553	7.4E-09	1.4E-08
	cg08285768	15	AKAP13*	0.27(2E-03)	-0.413	0.062	0.157	-0.829	3.8E-06	1.1E-05	-0.941	2.4E-11	5.3E-11
	cg06944982	8	PTK2*	0.33(2E-03)	-0.103	0.616	0.720	-0.761	4.1E-06	1.2E-05	-0.899	2.2E-10	4.5E-10
	cg23033749	7	ST7*	0.10(2E-03)	-0.160	0.045	0.136	-0.159	1.1E-02	1.6E-02	-0.205	3.9E-06	5.7E-06
	cg18999998	17	DHX8	0.39(2E-03)	-0.247	0.185	0.309	-0.482	2.4E-03	3.9E-03	-0.987	9.1E-07	1.4E-06
	cg03111404	6	GBD1;ZSCAN 31	0.20(2E-03)	-0.204	0.044	0.136	-0.463	5.9E-06	1.6E-05	-0.671	1.3E-16	4.7E-16
	cg16037200	15	MIR4510; CDIN1	0.19(2E-03)	-0.163	0.148	0.266	-0.743	4.2E-12	5.1E-11	-0.751	6.6E-18	2.7E-17
	cg01582980	7	SP8	0.30(4E-03)	-0.147	0.262	0.411	-0.628	2.1E-05	4.8E-05	-0.585	2.4E-07	3.8E-07

	cg09953520	8	*EBF3*	0.10(7E-03)	-0.149	0.054	0.151	-0.432	6.4E-08	2.8E-07	-0.670	3.5E-16	1.2E-15
	cg18379829	1	*MAP1LC3C; PLD5*	0.11(1E-02)	-0.134	0.137	0.259	-0.427	4.7E-07	1.8E-06	-0.571	1.1E-14	3.1E-14
	cg21879513	20	*COL20A1**	0.15(2E-02)	0.081	0.567	0.692	-0.313	2.0E-02	2.9E-02	-0.396	2.3E-04	2.9E-04
	cg26436731	1	*SPMIP3; ZBTB18**	0.10(2E-02)	-0.174	0.038	0.128	-0.606	7.0E-12	7.9E-11	-0.722	7.3E-20	3.8E-19
B	cg02133624	3	*DLG1*; DLG1-AS1*	0.16(1E-04)	-0.310	1.3E-06	4.0E-05	-0.294	1.3E-06	4.1E-06	-0.705	5.9E-23	5.3E-22
	cg04427437	3	*CNTN4*	0.20(6E-04)	-0.235	5.6E-03	2.6E-02	-0.243	6.9E-04	1.3E-03	-0.620	8.7E-21	5.6E-20
	cg19035181	20	*NINL*; NANP*; GINS1**	0.14(6E-04)	-0.307	1.4E-04	1.8E-03	-0.496	5.5E-10	4.0E-09	-0.885	2.1E-30	3.7E-29
	cg15889793	6	*MDGA1; AY927499*	0.18(9E-04)	-0.295	3.4E-04	2.9E-03	-0.361	9.2E-06	2.3E-05	-0.789	1.0E-24	9.7E-24
	cg11003536	11	*PRDM10*; LINC00167*	0.18(9E-04)	-0.341	2.0E-04	2.3E-03	-0.750	1.0E-15	2.3E-14	-0.983	1.7E-29	2.5E-28
	cg13632630	15	*LINC00052; NTRK3**	0.12(9E-04)	-0.174	4.2E-03	2.3E-02	-0.251	1.1E-05	2.7E-05	-0.497	5.5E-19	2.8E-18
	cg18777774	17	*ABR*; BHLHA9*	0.14(9E-04)	-0.524	3.7E-08	1.7E-06	-0.783	4.1E-17	1.1E-15	-1.209	3.9E-40	2.3E-38
	cg24836396	1	*ASH1L; MIR555*	0.18(1E-03)	-0.273	4.8E-03	2.4E-02	-0.623	2.4E-11	2.6E-10	-0.998	5.0E-28	6.9E-27
	cg26784162	2	*ATG9A*	0.13(1E-03)	-0.321	1.3E-05	2.5E-04	-0.674	6.5E-19	2.4E-17	-1.055	7.5E-36	2.3E-34
	cg01305421	12	*IGF1*	0.20(1E-03)	-0.325	3.1E-03	1.8E-02	-0.545	5.8E-07	2.1E-06	-0.875	1.6E-19	8.4E-19
	cg18223522	1	*FAM58B*	0.13(1E-03)	-0.317	3.2E-03	1.8E-02	-0.817	1.0E-17	3.1E-16	-1.034	2.6E-30	4.2E-29
	cg13556387	10	*ITGB1*	0.19(1E-03)	-0.248	4.8E-03	2.4E-02	-0.570	7.4E-11	7.4E-10	-0.652	5.4E-18	2.3E-17
	cg27401945	10	*VAX1; MIR3663HG*	0.20(2E-03)	-0.267	1.1E-02	4.6E-02	-0.375	1.4E-03	2.5E-03	-0.616	1.4E-11	3.2E-11
	cg08166720	17	*ZZEF1**	0.25(2E-03)	-0.410	1.1E-02	4.6E-02	-0.788	1.5E-09	9.9E-09	-1.192	1.1E-18	5.2E-18
	cg18909525	9	*ASB6**	0.30(2E-03)	-0.412	3.3E-05	5.3E-04	-0.330	2.5E-03	4.1E-03	-0.883	2.4E-15	7.1E-15
	cg00697880	3	*OSBPL10**	0.19(2E-03)	-0.366	1.0E-03	7.2E-03	-0.735	4.5E-13	6.7E-12	-1.033	5.4E-20	3.0E-19
	cg19452802	6	*CYP39A1; SLC25A27*	0.16(3E-03)	-0.310	1.4E-04	1.8E-03	-0.411	6.6E-06	1.8E-05	-0.618	4.6E-15	1.3E-14
	cg00335252	2	*RBMS1**	0.17(3E-03)	-0.422	2.6E-09	1.5E-07	-0.433	5.9E-09	3.4E-08	-0.891	7.0E-27	9.0E-26
	cg20931867	4	*MKRN9P; C12orf50*	0.34(3E-03)	-0.663	6.4E-03	2.9E-02	0.028	8.9E-01	9.1E-01	-0.575	5.0E-05	6.5E-05
	cg06772580	17	*TP53I14*	0.09(2E-02)	-0.275	9.8E-03	4.1E-02	-0.135	1.8E-01	2.2E-01	-0.425	1.3E-08	2.3E-08
C	cg08653258	3	*BHLHE40*; ARL8B**	-0.19(9E-04)	-0.253	0.013	0.051	-0.402	2.9E-08	1.5E-07	-0.737	3.1E-20	1.9E-19
	cg24408199	3	*EIF2B5*	-0.20(9E-04)	-0.049	0.665	0.758	-0.302	1.9E-03	3.2E-03	-0.302	1.8E-04	2.2E-04
	cg24595704	3	*EFHB; RAB5A*	-0.13(9E-04)	-0.126	0.055	0.151	-0.478	7.1E-13	9.8E-12	-0.521	1.8E-18	7.9E-18
	cg13273398	6	*RSPH4A*	-1.46(9E-04)	-0.661	0.305	0.457	-1.856	2.0E-02	2.9E-02	-0.726	1.6E-03	2.0E-03

	cg01533106	8	IQNK1; AX746851	-0.37(9E-04)	-0.097	0.499	0.628	-0.914	3.1E-09	1.9E-08	-0.799	3.4E-11	7.4E-11
	cg05452625	10	WNT8B	-0.46(9E-04)	-0.254	0.125	0.241	-0.527	4.3E-04	8.6E-04	-0.325	1.6E-02	1.9E-02
	cg17672798	10	ADARB2*	-0.17(9E-04)	-0.178	0.148	0.266	-0.485	5.8E-08	2.6E-07	-0.745	1.3E-21	8.4E-21
	cg20023354	12	ALG10	-0.23(9E-04)	-0.091	0.447	0.586	-0.348	1.6E-03	2.8E-03	-0.450	3.2E-07	5.1E-07
	cg26347606	12	ZNF26	-0.18(9E-04)	-0.173	0.088	0.195	-0.362	1.5E-05	3.6E-05	-0.289	2.7E-05	3.7E-05
	cg21533331	19	AC002116.7; THAP8; WDR62*	-0.19(9E-04)	-0.066	0.588	0.708	-0.220	3.2E-02	4.5E-02	-0.208	1.6E-03	1.9E-03
	cg08642843	10	STK32C; LRRC27	-0.28(9E-04)	-0.169	0.290	0.446	-0.568	5.0E-04	9.8E-04	-0.555	3.4E-06	5.1E-06
	cg13378886	1	STMN1	-0.22(1E-03)	-0.077	0.601	0.712	-0.683	7.0E-07	2.4E-06	-0.673	5.9E-15	1.6E-14
	cg18133284	3	CADPS; LINC00698	-0.16(1E-03)	-0.126	0.192	0.317	-0.383	1.0E-06	3.4E-06	-0.551	1.4E-16	4.9E-16
	cg20955022	7	SEMA3A	-0.18(1E-03)	-0.145	0.293	0.448	-0.528	2.7E-06	8.2E-06	-0.479	4.3E-10	8.5E-10
	cg08572940	19	ZNF121	-0.32(1E-03)	-0.079	0.759	0.823	-0.588	9.4E-03	1.4E-02	-0.350	4.2E-03	5.1E-03
	cg26261736	19	ZNF561-AS1; ZNF562	-0.15(1E-03)	-0.157	0.063	0.157	-0.520	2.7E-10	2.4E-09	-0.799	2.5E-25	2.6E-24
	cg00647046	10	INPP5A*; CFAP46	-0.34(1E-03)	0.055	0.772	0.829	-0.531	1.5E-03	2.6E-03	-0.661	1.4E-07	2.4E-07
	cg26328510	10	CUGBP2*	-0.56(1E-03)	-0.061	0.715	0.794	-1.054	3.4E-06	1.0E-05	-0.857	3.9E-06	5.7E-06
	cg16907075	1	UBQLN4	-0.90(1E-03)	-0.020	0.923	0.938	-0.768	2.6E-02	3.8E-02	-0.262	6.5E-02	7.3E-02
	cg20676475	1	LCE3D	-0.15(1E-03)	-0.190	0.050	0.145	-0.367	3.3E-05	7.4E-05	-0.551	1.6E-16	5.8E-16
	cg14575222	9	NAIF1; SLC25A25*	-0.18(2E-03)	-0.144	0.184	0.309	-0.479	3.3E-07	1.3E-06	-0.770	1.7E-18	7.8E-18
	cg16943505	11	LOC440040; OR4C13	-0.19(2E-03)	-0.113	0.497	0.628	-0.463	4.9E-04	9.5E-04	-0.619	5.4E-09	1.0E-08
	cg00336605	19	NR1H2	-0.22(2E-03)	0.072	0.638	0.741	-0.412	1.2E-03	2.1E-03	-0.429	1.2E-05	1.7E-05
	cg07177394	21	NCRNA00111	-0.13(2E-03)	-0.099	0.319	0.474	-0.385	2.2E-06	6.7E-06	-0.604	1.8E-17	6.8E-17
	cg15471815	1	HIST3H2BB; HIST3H2A	-0.27(2E-03)	0.142	0.452	0.586	-0.571	1.9E-03	3.2E-03	-0.281	4.2E-02	4.8E-02
	cg07074473	2	SNRNP28	-0.25(2E-03)	-0.069	0.729	0.805	-0.583	2.4E-04	5.0E-04	-0.188	1.3E-01	1.4E-01
	cg22279507	2	FARSB*	-0.30(2E-03)	-0.246	0.058	0.156	-0.739	5.2E-10	4.0E-09	-0.747	6.9E-11	1.5E-10
	cg04106641	2	ORC4L	-0.94(2E-03)	0.219	0.600	0.712	-1.165	5.7E-03	8.8E-03	-0.054	8.0E-01	8.2E-01
	cg19310148	7	NFE2L3	-0.27(2E-03)	-0.111	0.380	0.522	-0.370	2.1E-03	3.5E-03	-0.701	3.8E-12	8.8E-12
	cg13772903	14	C14orf19	-0.28(2E-03)	-0.408	0.070	0.168	-0.477	3.0E-03	4.8E-03	-0.294	3.5E-02	4.1E-02
	cg12741295	3	LRCH3	-0.30(2E-03)	-0.418	0.019	0.073	-0.971	3.3E-08	1.7E-07	-0.991	2.4E-15	7.1E-15
	cg14594063	10	ADAM12*	-0.27(2E-03)	-0.297	0.063	0.157	-0.412	1.9E-03	3.3E-03	-0.880	6.8E-14	1.8E-13
	cg00484532	8	PLEKHA2	-0.18(2E-03)	-0.181	0.042	0.133	-0.284	3.6E-03	5.7E-03	-0.561	3.9E-20	2.4E-19
	cg26034150	2	FAM126B; NDUFB3	-0.24(2E-03)	-0.301	0.113	0.224	-0.386	1.2E-02	1.8E-02	-0.692	2.2E-08	3.9E-08
	cg03964554	17	RAD51C*;	-0.17(3E-03)	0.085	0.395	0.535	-0.532	3.3E-09	2.0E-08	-0.281	5.8E-05	7.4E-05

			*PPM1E**										
	cg26319015	7	*ACTB*; FSCN1**	-0.28(3E-03)	-0.181	0.385	0.526	-0.788	3.0E-05	6.9E-05	-0.588	6.3E-06	9.1E-06
	cg18735146	13	*IFT88*	-0.13(3E-03)	-0.059	0.433	0.577	-0.390	5.1E-06	1.4E-05	-0.527	4.1E-12	9.3E-12
	cg27551227	7	*HTR5A*	-0.19(1E-02)	-0.252	0.054	0.151	-0.598	5.2E-07	1.9E-06	-0.427	1.7E-05	2.3E-05
	cg19086905	15	*ADAMTS17*	-0.18(1E-02)	-0.323	0.024	0.085	-0.418	1.1E-03	1.9E-03	-0.495	2.0E-06	3.0E-06
	cg05671758	16	*PIGQ*	-0.14(2E-02)	-0.137	0.359	0.513	-0.821	3.8E-08	1.9E-07	-0.765	5.8E-11	1.2E-10
	cg01832012	7	*TPK1**	-0.14(2E-02)	-0.153	0.083	0.192	-0.348	7.9E-06	2.0E-05	-0.354	5.9E-08	1.0E-07
	cg03029734	6	*GRIK2**	-0.12(5E-02)	0.008	0.928	0.938	-0.282	7.7E-04	1.4E-03	-0.252	1.4E-05	2.0E-05
D	cg08870757	17	*ALOX12**	-0.320(4E-04)	-0.451	2.5E-05	4.5E-04	-0.845	3.3E-15	6.0E-14	-0.820	1.7E-15	5.2E-15
	cg18771659	1	*SPRR1B*	-0.285(5E-04)	-0.311	2.3E-03	1.4E-02	-0.398	1.6E-04	3.4E-04	-0.537	5.6E-13	1.3E-12
	cg05053108	12	*GCN1L1*	-0.294(6E-04)	-0.536	3.7E-04	3.0E-03	-0.204	1.6E-01	2.0E-01	-0.324	1.9E-05	2.6E-05
	cg22789605	12	*SLC11A2**	-0.207(6E-04)	-0.433	3.4E-06	8.7E-05	-0.294	1.1E-03	2.0E-03	-0.573	9.3E-15	2.6E-14
	cg15210276	19	*HAPLN4**	-0.183(7E-04)	-0.306	4.3E-04	3.4E-03	-0.470	4.9E-08	2.2E-07	-0.715	1.3E-18	5.9E-18
	cg19681610	1	*NOS1AP**	-0.155(9E-04)	-0.290	1.3E-03	8.6E-03	-0.568	1.7E-12	2.2E-11	-0.768	3.4E-25	3.4E-24
	cg18190778	3	*PTPRG*	-0.252(9E-04)	-0.323	4.6E-03	2.4E-02	-0.469	7.3E-07	2.5E-06	-0.593	1.3E-12	3.1E-12
	cg08288130	8	*DOK2**	-0.255(9E-04)	-0.402	5.4E-05	8.1E-04	-0.506	5.5E-08	2.5E-07	-0.710	2.0E-15	6.2E-15
	cg11171549	11	*FXYD6*	-0.248(9E-04)	-0.267	3.2E-04	2.9E-03	-0.313	2.6E-05	6.0E-05	-0.500	1.0E-10	2.1E-10
	cg01788221	16	*ANKRD11**	-0.106(9E-04)	-0.392	1.3E-06	4.0E-05	-0.830	6.1E-28	1.1E-25	-1.039	4.1E-45	3.7E-43
	cg09043104	8	*LINC00536*; EIF3H**	-0.118(1E-03)	-0.338	8.1E-06	1.8E-04	-0.372	9.6E-07	3.2E-06	-0.650	1.9E-22	1.6E-21
	cg22933107	15	*AGBL1-AS1; AGBL1*	-0.132(1E-03)	-0.204	5.2E-03	2.5E-02	-0.352	1.3E-08	7.6E-08	-0.533	5.9E-20	3.2E-19
	cg25862345	21	*DSCR4*	-0.159(1E-03)	-0.295	4.4E-03	2.3E-02	-0.555	1.6E-08	8.5E-08	-0.752	3.8E-22	2.9E-21
	cg02052068	5	*UGT3A1*	-0.227(1E-03)	-0.451	2.2E-03	1.4E-02	-0.674	7.0E-08	2.9E-07	-1.002	4.1E-13	1.0E-12
	cg10063753	10	*EIF3A*	-0.318(1E-03)	-0.832	7.1E-16	1.3E-13	-1.491	8.2E-24	7.4E-22	-2.351	9.9E-50	1.8E-47
	cg00519039	10	*ARHGAP19*; FRAT1*	-0.201(2E-03)	-0.293	7.0E-03	3.1E-02	-0.476	5.2E-07	1.9E-06	-0.619	5.5E-13	1.3E-12
	cg19268446	19	*ZNF404*	-0.263(2E-03)	-0.355	7.7E-03	3.3E-02	-0.685	5.6E-10	4.0E-09	-0.742	1.6E-13	4.0E-13
	cg05834899	12	*SART3;ISCU*	-0.259(2E-03)	-0.454	1.9E-03	1.2E-02	-0.419	3.0E-03	4.8E-03	-0.594	4.9E-06	7.1E-06
	cg08647641	7	*CYP51A1*	-0.408(3E-03)	-0.776	1.6E-09	1.5E-07	-1.794	4.5E-19	2.0E-17	-2.999	3.5E-39	1.3E-37
	cg13676583	5	*DDX41*; DOK3**	-0.131(3E-03)	-0.200	1.3E-03	8.6E-03	-0.368	1.1E-09	7.3E-09	-0.476	9.0E-16	2.9E-15
	cg16419756	5	*SLC12A8**	-0.094(1E-02)	-0.224	2.6E-04	2.4E-03	-0.537	1.3E-15	2.6E-14	-0.736	1.1E-30	2.2E-29
	cg03940484	15	*SV2B*	-0.111(2E-02)	-0.302	2.5E-04	2.4E-03	-0.444	2.5E-07	1.0E-06	-0.591	3.1E-16	1.1E-15
	cg27196467	4	*KCNIP4*	-0.121(3E-02)	-0.335	2.5E-04	2.4E-03	0.003	9.7E-01	9.8E-01	-0.383	2.3E-08	4.0E-08
	cg01020987	1	*C1orf174; LOC1001337*	-0.097(3E-02)	-0.286	1.9E-04	2.3E-03	-0.273	2.1E-03	3.5E-03	-0.505	9.7E-14	2.5E-13

*Overlapped genes between our study and Lin et al., 2022
**Regression coefficients of DNAm at age 26 on parous status (yes/no) and cell-adjusted DNAm at age 18 and other covariates (see Chen et al., 2024 for more details)

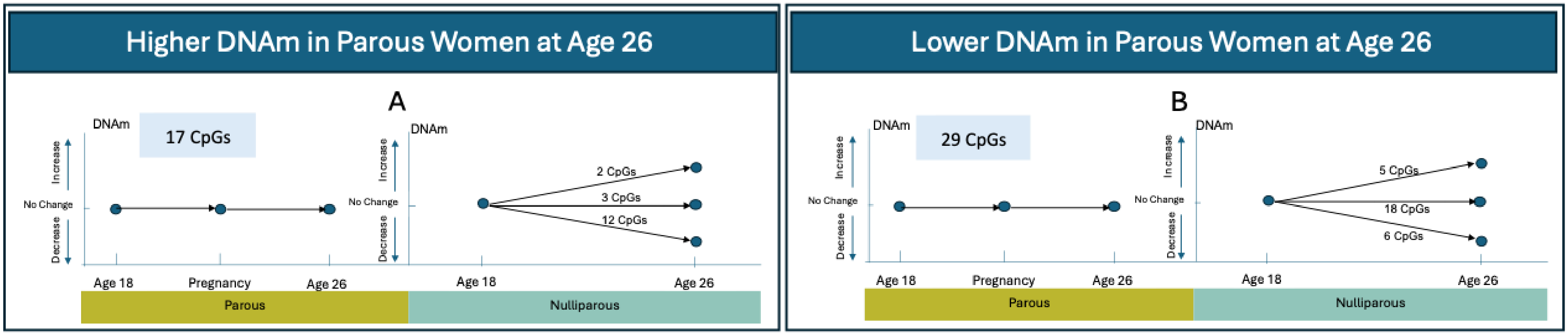

Figure S1: Conceptual display demonstrating no significant methylation changes over time in parous subjects. CpGs in panel A had higher DNAm in parous women at age 26, adjusted by DNAm at age 18 yr. CpGs in panel B had lower methylation in parous women at age 26, adjusted by DNAm at age 18 yr. Note that the magnitude of DNAm change is not depicted.

Table S2: Magnitude and significance indicators of all CpGs with DNA methylation that do not change across the timepoints of pre-pregnancy (age 18), gestation, and post-pregnancy (age 26) in parous subjects, and yet differ between parous and nulliparous subjects.

Group	CpG	Chr	Gene Name	Chen et al., 2024 (Parous vs Nulliparous)	Parous (Age 18 to Pregnancy)			Parous (Pregnancy to Age 26)			Nulliparous (Age 18 to Age 26)		
				Coef(FDRp)**	Coef	p-value	FDR-p	Coef	p-value	FDR-p	Coef	p-value	FDR-p
A	cg22894589	19	*TCF3**	0.42(6E-04)	-0.278	0.204	0.331	-0.346	0.101	0.131	-1.043	1.7E-13	4.3E-13
	cg13375690	8	*PDE7A*	0.24(9E-04)	-0.046	0.749	0.817	-0.217	0.127	0.160	-0.463	1.3E-05	1.8E-05
	cg07467765	17	*SEPTIN9; LINC01973*	0.31(9E-04)	-0.255	0.104	0.208	0.020	0.899	0.919	-0.848	4.0E-13	9.9E-13
	cg15509594	6	*FAM46A*	0.30(1E-03)	-0.283	0.130	0.249	-0.036	0.814	0.852	-0.586	7.4E-07	1.2E-06
	cg19138499	6	*REPS1*	0.39(1E-03)	0.116	0.445	0.586	-0.322	0.040	0.056	-0.600	8.6E-08	1.4E-07
	cg02153334	5	*TERT*	0.40(1E-03)	-0.134	0.546	0.678	-0.291	0.134	0.167	0.006	9.7E-01	9.7E-01
	cg17078116	8	*NEFM*	0.25(1E-03)	-0.010	0.948	0.953	0.024	0.841	0.870	-0.024	8.0E-01	8.2E-01
	cg07624483	11	*TMEM132A*	0.48(2E-03)	-0.052	0.862	0.902	-0.293	0.211	0.246	-0.548	8.0E-03	9.5E-03
	cg19135706	5	*RAB3C*	0.32(2E-03)	-0.403	0.078	0.181	-0.121	0.524	0.576	-0.846	2.8E-09	5.2E-09
	cg16710348	3	*SLC15A2; SLC15A3*	0.31(2E-03)	-0.274	0.171	0.295	-0.354	0.042	0.057	-0.673	1.9E-06	2.8E-06

	cg23243867	1	ST6GALNAC5	0.54(3E-03)	-0.410	0.168	0.295	-0.047	0.918	0.933	-1.276	5.0E-09	9.4E-09
	cg11704005	1	PAX7	0.13(7E-03)	-0.031	0.736	0.808	0.107	0.206	0.242	0.184	4.6E-03	5.5E-03
	cg25107893	1	C1orf170	0.14(9E-03)	0.354	0.036	0.125	0.218	0.198	0.235	0.671	1.7E-10	3.5E-10
	cg00271807	2	FAR2P1	0.17(1E-02)	-0.422	0.041	0.131	-0.412	0.056	0.076	-0.692	3.4E-07	5.3E-07
	cg07455279	19	NDUFA4	0.16(1E-02)	-0.221	0.059	0.157	-0.217	0.112	0.142	-0.353	2.3E-05	3.1E-05
	cg07835443	16	SPATA33	0.10(2E-02)	-0.061	0.516	0.645	-0.126	0.207	0.242	-0.403	2.0E-09	3.9E-09
	cg20464719	19	FCHO1*	0.17(2E-02)	0.035	0.826	0.870	0.321	0.046	0.062	0.162	8.5E-02	9.5E-02
B	cg24028350	3	UBP1*	-0.27(5E-04)	-0.241	0.223	0.356	0.064	0.674	0.710	0.013	8.6E-01	8.8E-01
	cg17356328	12	FBXL14	-0.56(5E-04)	-0.420	0.151	0.269	0.441	0.075	0.101	0.229	9.5E-02	1.0E-01
	cg19611175	3	CCDC48	-0.33(6E-04)	0.402	0.019	0.073	-0.200	0.171	0.207	0.004	9.6E-01	9.7E-01
	cg22434409	4	KCNIP4	-0.23(6E-04)	-0.170	0.203	0.331	0.061	0.554	0.597	0.366	1.4E-07	2.4E-07
	cg04318212	14	MAX	-0.18(9E-04)	-0.173	0.092	0.199	-0.155	0.098	0.128	-0.273	1.6E-05	2.3E-05
	cg13629270	20	CHD6*	-0.33(9E-04)	0.323	0.173	0.297	-0.223	0.255	0.292	0.006	9.7E-01	9.7E-01
	cg22105146	4	TMEM165	-0.30(9E-04)	-0.223	0.356	0.512	0.313	0.092	0.121	0.463	8.8E-04	1.1E-03
	cg21008928	4	GBA3	-0.29(9E-04)	-0.302	0.092	0.199	-0.127	0.413	0.465	-0.262	3.4E-02	4.0E-02
	cg13334788	3	NAKCNH8; EFHB	-0.16(1E-03)	0.127	0.045	0.136	-0.039	0.514	0.568	0.207	8.2E-06	1.2E-05
	cg04741861	19	FZR1*	-0.52(1E-03)	-0.266	0.273	0.424	-0.005	0.979	0.984	0.205	2.0E-01	2.2E-01
	cg22340644	7	HIP1*	-0.15(1E-03)	-0.173	0.146	0.266	-0.050	0.621	0.658	-0.296	4.1E-08	7.0E-08
	cg03119897	1	NFASC*	-0.36(1E-03)	0.081	0.652	0.751	-0.034	0.820	0.853	0.078	4.5E-01	4.8E-01
	cg21931792	8	PXMP3	-0.19(1E-03)	-0.218	0.104	0.208	-0.156	0.163	0.202	-0.322	4.3E-05	5.6E-05
	cg12439527	15	MAN2C1	-0.37(1E-03)	-0.216	0.344	0.504	-0.247	0.178	0.215	0.184	2.3E-01	2.5E-01
	cg24858279	22	CBX6*	-0.33(2E-03)	-0.014	0.926	0.938	0.130	0.393	0.445	0.363	4.3E-05	5.7E-05
	cg08413026	12	ULK1*	-0.37(2E-03)	-0.005	0.982	0.982	-0.123	0.539	0.585	0.436	6.0E-03	7.2E-03
	cg20254483	10	UNC5B*	-0.43(2E-03)	0.196	0.687	0.776	-0.176	0.605	0.644	0.406	5.1E-02	5.8E-02
	cg11697983	1	SERBP1	-0.23(2E-03)	-0.163	0.553	0.682	0.195	0.339	0.386	0.105	4.6E-01	4.9E-01
	cg06944092	19	APLP1	-0.27(2E-03)	-0.156	0.371	0.521	-0.081	0.535	0.584	-0.121	2.5E-01	2.7E-01
	ch.17.71764510F	17	LINC00469; LINC02092	-0.49(2E-03)	0.459	0.221	0.355	-0.468	0.110	0.142	-0.350	6.0E-02	6.8E-02
	cg07369363	12	C12orf35	-0.20(2E-03)	-0.021	0.878	0.912	-0.068	0.557	0.597	-0.232	4.8E-02	5.6E-02
	cg18866106	6	ZKSCAN3*; ZNF323	-0.40(2E-03)	0.149	0.694	0.776	0.222	0.461	0.512	-0.096	6.3E-01	6.6E-01
	cg03711182	15	RASGRF1*	-0.34(3E-03)	-0.481	0.076	0.179	-0.320	0.112	0.142	-0.109	4.4E-01	4.7E-01
	cg11474763	8	HSF1	-0.17(4E-03)	-0.147	0.049	0.145	-0.124	0.083	0.110	-0.089	2.1E-01	2.3E-01
	cg21567066	4	KIAA0922; KIAA0923	-0.21(6E-03)	0.166	0.379	0.522	-0.118	0.426	0.476	0.049	6.3E-01	6.6E-01
	cg13925686	17	ACAP1*	-0.19(7E-03)	-0.071	0.494	0.628	-0.169	0.184	0.220	-0.375	8.0E-06	1.1E-05
	cg27318352	16	PPP4C	-0.80(2E-02)	0.144	0.590	0.708	-0.002	0.996	0.996	0.014	9.3E-01	9.4E-01
	cg01537571	15	TM2D3	-0.30(2E-02)	0.043	0.916	0.938	-0.562	0.169	0.207	-0.273	6.3E-02	7.1E-02
	cg19930135	2	GMPPA	-0.16(4E-02)	-0.157	0.069	0.168	-0.179	0.075	0.101	-0.447	1.0E-06	1.6E-06

* Overlapped genes between our study and Lin et al., 2022.

** Regression coefficients of DNAm at age 26 on parous status (yes/no) and cell-adjusted DNAm at age 18 and other covariates (see Chen et al., 2024 for more details)

Table S3: KEGG biological pathway and biological process associated with 112 overlapped genes between Lin et al., 2022 and Gruzieva et al., 2019 or Lin et al., 2022 and Fradin et al., 2023

**KEGG Biological Pathway**	**p-value**	**q-value FDR B&H**	**Hit Count in Query (Hit Count in Genome)**	**Hits in the Query List**
Medicus pathogen HPV E6 to notch signaling pathway	4.17E-05	0.005	3 (12)	JAG1,MAML2,NOTCH1
**Biological Process**	**p-value**	**q-value FDR B&H**	**Hit Count in Query (Hit Count in Genome)**	**Hits in the Query List**
Regulation of fat cell differentiation	2.51E-06	0.007	8 (168)	ZBTB16,JDP2,MECOM,SH3PXD2B,PIM1,JAG1,RORC,PRDM16
Fat cell differentiation	1.94E-05	0.025	9 (291)	ZBTB16,JDP2,MECOM,SH3PXD2B,PIM1,JAG1,ARID5B,RORC,PRDM16
Positive regulation of signal transduction	4.31E-05	0.025	23 (1824)	RPL26,TRIM15,TNFSF10,TRIM26,NTRK3,MAP3K20,GRB10,CHSY1,SPECC1L,GCNT2,ZEB2,MIR10A,TXK,LIMS1,KANK1,PTPRJ,NACC2,PIM1,CSNK1D,JAG1,RNF146,CLEC16A,NOTCH1
Hemopoiesis	4.83E-05	0.025	18 (1223)	ZBTB16,MYO1E,MECOM,RUNX1,INPP4B,SBNO2,TXK,SART1,HOXB3,HOXB4,PTPRJ,PIM1,JAG1,STON2,RORC,UBASH3B,PRDM16,NOTCH1
Positive regulation of fat cell differentiation	5.43E-05	0.025	5 (79)	ZBTB16,MECOM,SH3PXD2B,PIM1,PRDM16
Positive regulation of cell differentiation	6.92E-05	0.025	17 (1141)	RIPOR2,ZBTB16,NTRK3,MECOM,GCNT2,RUNX1,ZEB2,SOCS2,SART1,SLC6A6,HOXB4,SH3PXD2B,PIM1,JAG1,PRDM16,CDH4,NOTCH1
Hematopoietic stem cell proliferation	6.97E-05	0.025	4 (43)	MECOM,RUNX1,HOXB4,PIM1
Connective tissue development	6.98E-05	0.025	9 (343)	ZBTB16,CHSY1,RUNX1,BMP1,HOXB3,SH3PXD2B,ARID5B,RORC,NOTCH1
Regulation of cell adhesion	7.80E-05	0.025	15 (927)	RIPOR2,ZBTB16,SPECC1L,GCNT2,RUNX1,MIR10A,SART1,LIMS1,KANK1,PTPRJ,JAG1,TREM1,UBASH3B,NOTCH1,APBB1IP
Positive regulation of brown fat cell differentiation	1.02E-04	0.027	3 (18)	MECOM,PIM1,PRDM16
Neutrophil extravasation	1.02E-04	0.027	3 (18)	RIPOR2,JAML,TREM1
Negative regulation of cell adhesion	1.37E-04	0.032	9 (375)	RIPOR2,SPECC1L,GCNT2,RUNX1,MIR10A,KANK1,JAG1,UBASH3B,NOTCH1
Regulation of cell differentiation	1.44E-04	0.032	23 (1974)	RIPOR2,ZBTB16,NTRK3,JDP2,MECOM,GCNT2,RUNX1,ZEB2,MIR10A,INPP4B,SOCS2,SART1,SLC6A6,HOXB3,HOXB4,SH3PXD2B,PIM1,JAG1,RORC,UBASH3B,PRDM16,CDH4,NOTCH1

Table S4: Top 10 diseases associated with 112 overlapped genes between Lin et al., 2022 and Gruzieva et al., 2019 or Lin et al., 2022 and Fradin et al., 2023

**Disease Name**	**p-value**	**q-value FDR B&H**	**Hit Count in Query (Hit Count in Genome)**	**Hits in the Query List**
Leukocyte count	3.13E-11	6.05E-08	29 (1913)	VMP1,ZBTB16,DDAH1,MYO1E,ZNF366,RAP1GAP2,CHSY1,JDP2,MECOM, SPECC1L,GCNT2,RUNX1,CHI3L2,PCGF3,JAML,SBNO2,ITPK1,MGAT3,TULP4,PTPRJ,LINC00880,JAG1,TBC1D14,RERE,UBASH3B,CLEC16A,FHIP1A,KDM4B,APBB1IP
Monocyte percentage of leukocytes	1.75E-10	1.69E-07	18 (731)	VMP1,ZBTB16,TNFSF10,CHSY1,MECOM,ARHGAP9,ST3GAL3,GCNT2,RUNX1,PCGF3,CSNK1D,TBC1D14,MLLT1,RERE,UBASH3B,PRDM16,KDM4B,APBB1IP
Platelet crit	1.11E-08	7.15E-06	18 (952)	VMP1,ZBTB16,MYO1E,MYO9B,MECOM,RUNX1,ITPK1,SERPINA1,TULP4,LINC00880,STON2,ARID5B,RERE,UBASH3B,DNAJC5B,PRDM16,FHIP1A,RCAN2
Appendicular lean mass	1.51E-08	7.32E-06	23 (1607)	VMP1,DDAH1,MYO1E,MYO9B,GRB10,CHSY1,JDP2,MECOM,RABGAP1,RUNX1,PCGF3,ADCY3,ITPK1,PDLIM4,SOCS2,SERPINA1,MGAT3,TULP4,LINC00880,JAG1,ARID5B,RERE,FHIP1A
Neutrophil percentage of leukocytes	5.27E-08	1.74E-05	14 (610)	RIPOR2,ZBTB16,ZNF366,RAP1GAP2,CHSY1,JDP2,PCGF3,JAML,TBC1D14, ARID5B,RERE,UBASH3B,CLEC16A,KDM4B
Myeloid white cell count	5.39E-08	1.74E-05	17 (937)	VMP1,ZBTB16,MYO1E,ZNF366,CHSY1,MECOM,GCNT2,CHI3L2,PCGF3,JAML,PTPRJ,LINC00880,TBC1D14,RERE,UBASH3B,DNAJC5B,APBB1IP
Lymphocyte count	6.88E-08	1.90E-05	21 (1464)	VMP1,RIPOR2,ZBTB16,DDAH1,RAP1GAP2,JDP2,PFKFB3,GCNT2,INPP4B, SBNO2,ITPK1,PDLIM4,PTPRJ,JAG1,TBC1D14,ARID5B,RERE,UBASH3B,CLEC16A,KDM4B,APBB1IP
Neutrophil count, eosinophil count	9.48E-08	2.29E-05	9 (213)	VMP1,ZBTB16,MYO1E,ZNF366,CHSY1,PCGF3,JAML,PTPRJ,RERE
Monocyte count	3.02E-07	6.48E-05	19 (1320)	VMP1,ZBTB16,MYO1E,TNFSF10,ZNF366,RAP1GAP2,MECOM,ARHGAP9,GCNT2,RUNX1,JAML,ITPK1,LINC00880,JAG1,TBC1D14,MLLT1,RNF216,UBASH3B,KDM4B
Platelet count	7.09E-07	1.37E-04	21 (1686)	ZBTB16,MYO9B,MECOM,RUNX1,CDC42BPB,PHGDH,SBNO2,ITPK1,SERPINA1,LIMS1,PTPRJ,PIM1,LINC00880,TBC1D14,STON2,ARID5B,RERE,DNAJC5B,PRDM16,FHIP1A,RCAN2
